# HLA-DRB1*15:01 drives sex- and age-dependent microglial immune phenotypes and neuroimmune signaling

**DOI:** 10.3389/fimmu.2026.1796692

**Published:** 2026-06-16

**Authors:** Elsa M. Reyes-Reyes, Dhanalakshmi Chinnasamy, Michael D. Trial, Fernando Fernandez, Vincent D. Nguyen, Qianying He, Angel-Grace Leslie, Jessica Begay, Jessica Triplett, David Bradford, Carolina Figueroa, Jean P. Wiegand, Kathleen E. Rodgers

**Affiliations:** 1 Center for Innovation in Brain Science, University of Arizona, Tucson, AZ, United States; 2 Clinical Translational Science, University of Arizona, Tucson, AZ, United States; 3 Dinè College, Tsaile, AZ, United States; 4 Department of Pharmacology, College of Medicine, University of Arizona, Tucson, AZ, United States

**Keywords:** aging, HLA-DRB1*15:01, microglial activation, neuroimmune signaling, neuroinflammation, sex differences

## Abstract

**Introduction:**

The major histocompatibility complex class II (MHC-II) pathway is central to adaptive immunity and immune tolerance, and its age-related dysregulation is increasingly linked to chronic neuroinflammation. The HLA-DRB1*15:01 allele, the strongest genetic risk factor for multiple sclerosis, has been implicated in shaping pathogenic CD4^+^ T-cell responses and broader neuroimmune vulnerability, yet how this allele modulates age- and sex-dependent neuroimmune processes within the central nervous system (CNS) remains poorly defined.

**Methods:**

We investigated the impact of HLA-DRB1*15:01 expression using a humanized mouse model (HLA mice) and wild-type (WT) controls. Male and female mice were analyzed at 6, 9, and 15 months of age, with endocrine stratification in females. Behavioral testing, flow cytometry, immunofluorescence, and multiplex cytokine analyses were used to assess cognitive performance, glial immune-associated changes and oxidative stress, astrocyte–microglia IL-3/IL-3R signaling, endothelial activation, selective immune cell accumulation at CNS borders, tissue organization, and hippocampal cytokine profiles.

**Results:**

HLA mice developed age- and sex-dependent cognitive impairment, most pronounced in aged females. HLA-DRB1*15:01 expression promoted progressive microglial immune-associated changes, characterized by increased CD14 and CD68 expression, elevated mitochondrial oxidative stress, altered astrocyte phenotypes, and enhanced IL-3/IL-3R signaling. Hippocampal axonal and myelin organization was disrupted in aged HLA mice and was spatially associated with increased microglial presence. HLA mice also exhibited selective immune remodeling, including increased accumulation of CD4^+^ T cells and NK1.1^+^CD3^+^ natural killer T (NKT) cells, particularly in females, accompanied by endothelial activation marked by elevated ICAM-1 and E-selectin expression. Hippocampal cytokine profiling revealed selective sex-biased alterations, without broad induction of classical inflammatory cytokines.

**Conclusion:**

Together, these findings demonstrate that HLA-DRB1*15:01 drives a coordinated, age- and sex-dependent neuroinflammatory program linking behavioral dysfunction, glial immune-associated changes and oxidative stress, selective immune cell recruitment, endothelial activation, tissue remodeling, and targeted cytokine imbalance. This integrated phenotype provides mechanistic insight into how this major MS risk allele confers vulnerability to chronic neuroinflammation during aging, with heightened impact in females, independent of reproductive cycling stage.

## 1 Introduction

The major histocompatibility complex class II (MHC-II) pathway plays a central role in adaptive immunity by enabling antigen-presenting cells (APCs) to present extracellular peptides to CD4^+^ T lymphocytes. During early life, presentation of self-antigens plays a critical role in establishing central and peripheral immune tolerance by eliminating or functionally inactivating autoreactive T cells through thymic deletion, induction of anergy, and suppression by regulatory T cells. With aging, however, tolerance mechanisms progressively decline, driven by reduced regulatory T-cell function, altered sensitivity of effector T cells to suppressive signals, and cumulative inflammatory exposure. This age-associated erosion of immune tolerance may permit the activation of previously restrained autoreactive T cells, thereby promoting chronic, low-grade neuroinflammation (1, 2).

Human leukocyte antigen (HLA) class II molecules—including HLA-DR, HLA-DQ, and HLA-DP—are highly polymorphic, and variation within these loci represents a major genetic determinant of autoimmune disease susceptibility. Among these, polymorphisms in HLA-DRB1 have been most consistently linked to immune-mediated disorders, underscoring the importance of antigen presentation in shaping pathogenic CD4^+^ T-cell responses (3–5). The HLA-DRB1*15:01 allele is the strongest and most reproducible genetic risk factor for multiple sclerosis (MS), conferring approximately a 3-fold increase in disease susceptibility (6). Beyond MS, HLA-DRB1*15:01 has been associated with several other autoimmune conditions, including juvenile idiopathic arthritis, Sjögren’s syndrome, and systemic lupus erythematosus. Many of these disorders exhibit cognitive dysfunction or central nervous system (CNS) involvement, suggesting that HLA-DRB1*15:01–associated immune dysregulation is not restricted to peripheral autoimmunity (7–13). Moreover, emerging genetic and epidemiological studies implicate variation within the HLA-DRB1*15–01 haplotype in susceptibility to late-onset neurodegenerative diseases, including Alzheimer’s disease (14–16) and Parkinson’s disease (17, 18), pointing to a broader role for this allele in age-related neuroimmune vulnerability. Mechanistically, HLA-DRB1*15:01 shapes the repertoire, activation state, and cytokine polarization of CD4^+^ T cells through selective presentation of self and foreign peptides (17, 19, 20). In MS lesions, HLA-DRB1*15:01 molecules have been shown to present myelin basic protein (MBP)–derived epitopes *in situ*, and structural studies demonstrate stable binding of the immunodominant MBP_85–99_ peptide within the HLA-DRB1*15:01 peptide-binding groove (21, 22). HLA-DRB1*15:01 can also bind peptides derived from proteins associated with neurodegeneration, including amyloid-β (15) and α-synuclein (17), suggesting that this allele contributes to immune recognition of neuronal and glial self-antigens under permissive inflammatory conditions. These findings provide direct molecular evidence linking this allele to autoreactive CD4^+^ T-cell responses within the CNS. Despite the well-established role of HLA-DRB1*15:01 in shaping CD4^+^ T-cell responses (17, 19, 20), how this allele influences age- and sex-dependent neuroimmune interactions within the CNS remains poorly defined. In particular, it is unclear how HLA-DRB1*15:01 expression modulates resident glial states, neuroimmune signaling pathways, immune–CNS interfaces, and tissue-level organization during aging in the absence of overt autoimmune challenge. To isolate HLA-DRB1*15:01–specific mechanisms *in vivo*, we used a previously established transgenic mouse model generated on an MHC-II–null background that expresses the human HLA-DRB1*15:01 peptide-binding domains under the control of the murine I-E promoter (22). This design ensures physiological, antigen-presenting cell–restricted expression of HLA-DRB1*15:01 and supports normal CD4^+^ T-cell development and function. These mice develop chronic experimental autoimmune encephalomyelitis (EAE) following immunization with myelin oligodendrocyte glycoprotein, establishing that HLA-DRB1*15:01–restricted CD4^+^ T cells are sufficient to drive CNS autoimmunity *in vivo* (22). However, the impact of HLA-DRB1*15:01 expression on spontaneous, age-related neuroinflammatory processes, independent of induced autoimmunity, has not been systematically examined. In the present study, we investigated how HLA-DRB1*15:01 expression shapes neuroimmune aging by integrating behavioral, cellular, molecular, and tissue-level analyses in male and female mice across multiple ages. We examined microglial oxidative stress and immune-associated changes, astrocyte–microglia cytokine signaling, endothelial activation, immune cell accumulation at CNS interfaces, hippocampal cytokine profiles, and associated behavioral outcomes. Together, these studies define how a major human autoimmune risk allele intersects with aging and sex to remodel the neuroimmune environment, providing mechanistic insight into how genetic susceptibility may promote chronic neuroinflammation and CNS vulnerability over the lifespan.

## 2 Material and methods

### 2.1 Animals and brain cells isolation

All animal studies and procedures were conducted in accordance with the National Institutes of Health guidelines for the care and use of laboratory animals and were approved by the University of Arizona Institutional Animal Care and Use Committee (IACUC). Female and male HLA-DRB1*15:01 transgenic mice were obtained from Dr. Vandenbark; the generation and characterization of this mouse strain have been described previously. Age-matched B6D2F1/J wild-type mice were obtained from The Jackson Laboratory (JAX:100006) at 12 weeks of age. All mice were bred and/or aged in a health-designated (HD) animal facility under specific-pathogen-free conditions (Helicobacter- and murine norovirus–negative), with non-sterile food and housing. Reproductive/endocrine aging status was assessed in female HLA-DRB1*15:01 mice at 6, 9, and 15 months of age by monitoring estrous cyclicity using daily vaginal cytology for 3 consecutive weeks. The cycle stage was determined from the predominant cellular composition observed in vaginal smears, including leukocytes, nucleated epithelial cells, and cornified epithelial cells. Based on established cytological criteria, animals were classified into proestrus, estrus, metestrus, or diestrus stages (23). Females were categorized according to cycling pattern as regular cycling (4–5 day cycles), irregular cycling (6–9 day cycles), or acyclic (absence of cycling for ≥9 days) to evaluate age-associated reproductive transition. Mice were anesthetized with isoflurane at 6, 9, or 15 months of age, and blood was collected by cardiac puncture. Animals were then perfused transcardially with cold phosphate-buffered saline (PBS; Research product international, #P32080-100T), and brains were harvested. Brain tissue was dissociated into single-cell suspensions using the Miltenyi Biotec Adult Brain Dissociation Kit (Cat. no. 130-107-677) according to the manufacturer’s instructions.

### 2.2 Nesting test

Nestlet shredding and nest construction were assessed as measures of spontaneous, goal-directed home-cage behavior (24). Mice were individually housed for 24 hours in clean cages containing ~1 cm of Alpha-Dri bedding and provided with a single 5 × 5 cm compressed cotton Nestlet (Ancare, USA, # NES3600), with ad libitum access to food and water. Cages were labeled to blind the experimenter to genotype and experimental group. After 18 hours, cages were inspected and overhead photographs were obtained. Nesting behavior was scored using a standardized 1–5 scale adapted from established protocols, in which scores reflected the extent of Nestlet shredding and nest structure: 1 = Nestlet largely intact (>90% intact); 2–3 = partial to extensive shredding without a consolidated nest site; and 4–5 = formation of an identifiable to near-perfect crater-shaped nest with shredded material gathered into a defined structure. Scoring was performed by an experimenter blinded to experimental conditions.

### 2.3 Novel object recognition test

Intermediate memory was assessed using the novel object recognition (NOR) test (25, 26). Mice were first given a 24-hour habituation and familiarization period to acclimate to the testing environment and a set of identical familiar objects. NOR testing was conducted in arenas measuring 32 × 26 × 30 cm, with up to eight arenas prepared simultaneously. Two identical familiar objects were placed diagonally within each arena, equidistant from the arena walls. During the familiarization phase, each mouse was allowed to freely explore the arena and the two familiar objects for 15 minutes. Twenty-four hours later, the test phase was conducted by replacing one of the familiar objects with a novel object positioned in the same location and orientation as the original familiar object. Mice were again allowed to explore the arena for 15 minutes. Behavior during the test phase was recorded using an overhead camera and analyzed with an automated tracking system (AnyMaze software, Stoelting Co.). The software tracked each mouse throughout the test session and quantified the time spent exploring the novel and familiar objects, defined as directed head orientation toward the object. Exploration times were used to calculate the following indices: Recognition Index (%) = 100 × [novel object exploration time/(novel + familiar object exploration time)] and Discrimination Index (%) = 100 × [(novel object exploration time − familiar object exploration time)/(novel + familiar object exploration time)].

### 2.4 Immunohistochemistry

Immunohistochemistry was performed as previously described (4). Briefly, brain hemispheres were fixed overnight in 4% paraformaldehyde (Thermo Fisher Scientific, #J19943-K2) at 4 °C and subsequently transferred to 30% sucrose (Millipore Sigma #S9378) for cryoprotection. Tissue was embedded in optimal cutting temperature (OCT) (VWR, #25608-930) compound and sectioned using a cryostat. Coronal sections (15 µm thickness) were collected consecutively along the anterior (rostral) to posterior (caudal) axis of the brain to preserve regional continuity. Sections containing the hippocampus were selected for analysis of the dentate gyrus region. Four sections per mouse were processed and quantified, with one image acquired per section. A total of four mice per experimental group were analyzed, using one hemisphere from each brain. Sections were blocked for 60 minutes at room temperature in 0.5% Triton X-100 (Millipore Sigma #X100) in PBS containing 5% normal goat serum (NGS) (Thermo Fisher Scientific, #16210072). Primary antibodies were diluted in 0.5% Triton X-100/PBS with 5% NGS and applied overnight at 4 °C (rabbit anti-MBP (1:500, Abcam, #Ab40390), rabbit anti-IL-3R (1:200, Bioss, #BS-2600R), rat anti-IL-3 (1:500, Biolegend, #503902), rat anti-CD68 (1:200, Thermo Fisher Scientific, #14-0681-82), mouse anti-NF-200 (1:500, Thermo Fisher Scientific, #13-1300), chicken anti-GFAP (1:1000, Abcam, # Ab4674), guinea pig anti-IBA1 (1:1000, Synaptic systems, #HS234 308)). Sections were then thoroughly washed in 0.5% Triton X-100/PBS and incubated for 1 hour at room temperature with the appropriate secondary antibodies (#Ab150169, #Ab150078, #Ab150159 from Abcam and #A-11073, #A-11077, #A-21244, #A-21235 from Thermo Fisher Scientific), diluted 1:1000 in the same blocking solution. Following washing, sections were mounted onto glass slides using a DAPI-containing mounting medium (VectaShield, #H-1200-10) and coverslipped. Immunofluorescent images were acquired using a Zeiss LSM 880 NLO confocal microscope at 20× magnification. Quantification of microglial branch number and process length was performed using Fiji (ImageJ), while immunoreactivity and colocalization analyses were performed using QuPath.

### 2.5 Flow cytometry staining

Single-cell suspensions prepared from mouse brain tissue were used for multiparameter flow cytometry analyses. All staining procedures were performed using ice-cold buffers unless otherwise indicated, and samples were protected from light throughout. Prior to staining with cell-surface marker antibodies, cells were incubated with an Fc receptor–blocking reagent (Miltenyi Biotec, #130-092-575) for 15 minutes at 4 °C to prevent nonspecific antibody binding. For assessment of oxidative stress, cells were incubated with 5 µM MitoSOX™ Red (Thermo Fisher Scientific, #M36008) in DPBS supplemented with glucose (Thermo Fisher Scientific, #14287-072) at 37 °C for 20 minutes in the absence of CO_2_. Cells were then stained with cell-surface marker antibodies for 25 minutes at 4 °C, washed with PBS, and centrifuged at 400 × g for 5 minutes. For intracellular staining, cells were fixed using eBioscience™ IC Fixation Buffer (Thermo Fisher Scientific, #00-8222-49) for 15 minutes, followed by permeabilization with eBioscience™ Permeabilization Buffer (Thermo Fisher Scientific, #00-8333-56) for 20 minutes. After washing, cells were incubated with antibodies recognizing intracellular proteins for 25 minutes at room temperature. Cell pellets were resuspended in PBS for acquisition. For unfixed samples, DAPI (Miltenyi Biotec, #130-111-570) was added immediately prior to data acquisition to exclude dead cells. Samples were analyzed by flow cytometry using a MACSQuant 10 (Miltenyi Biotec), and data were analyzed with FlowLogic software.

### 2.6 Flow cytometry cell population definitions

Antibody combinations were selected to enable accurate identification of specific immune and glial cell populations and their functional states based on established marker expression. Antibody details are provided in **Supplementary Table 1**. Microglia were defined as CD45 ^low/int^ CD11b^+^ Ly-6C^-^ cells, where CD45 was used to identify leukocytes, CD11b identified the myeloid lineage, and Ly-6C was used to exclude infiltrating monocytes. Within the microglial population, activation- and stress-associated markers were quantified, including CD14 (innate immune activation–associated receptor), CD68 (lysosomal/phagolysosomal marker indicative of microglial activation), CD123 (IL-3Rα, IL-3 receptor expression), and MitoSOX™ Red (mitochondrial superoxide production). Astrocytes were identified by surface expression of ACSA-2. Intracellular staining was used to assess astrocytic functional markers, including IL-3, an astrocyte-derived cytokine, and GFAP, an intermediate filament protein associated with astrocytic structure and reactivity. T-cell populations were identified using CD45 to define leukocytes and CD3ϵ to identify T-cell lineage. T-cell subsets were further distinguished by expression of CD4 (helper T cells) and CD8β (cytotoxic T cells). NK1.1 expression in combination with CD3ϵ was used to identify NK1.1^+^CD3ϵ^+^ natural killer T (NKT) cells. Endothelial cells were identified by surface expression of CD31 (PECAM-1), a pan-endothelial marker. Endothelial activation status was assessed by measuring the expression of adhesion molecules involved in leukocyte tethering, adhesion, and transmigration, including CD54 (ICAM-1), CD106 (VCAM-1), CD62E (E-selectin), and CD62P (P-selectin). ICAM-1 and VCAM-1 were evaluated as indicators of firm leukocyte adhesion, whereas E-selectin and P-selectin were assessed as markers of endothelial activation associated with leukocyte rolling and recruitment at the neurovascular interface.

### 2.7 Flow cytometry gating strategy

For brain flow cytometry analyses, the total number of acquired events ranged from approximately 317,000 to >488,000 per sample, followed by sequential gating for singlets, live cells, and downstream immune and glial populations. Representative acquisition depth and downstream event counts for the principal gated populations analyzed in this study are provided in **Supplementary Table 2**. The sequential gating strategies used to identify these populations using Flow Logic software are illustrated in **Supplementary Figures 1**-**5**. For all panels, cells were first gated on forward scatter (FSC-A) versus side scatter (SSC-A) to exclude debris. Dead cells were excluded using DAPI as indicated. Doublets were removed using FSC-A versus FSC-H gating.

Panel 1: Microglia identification and mitochondrial superoxide. Microglia were identified as CD11b^+^ CD45^low/int^ cells, with peripheral myeloid cells excluded based on Ly6C expression. Mitochondrial superoxide production was assessed using MitoSOX-PE, with fluorescence quantified within the gated microglial population (**Supplementary Figure 1**).

Panel 2: Microglial immune-associated changes. Using the same microglial gating strategy for microglia identification, activation status was evaluated by analysis of CD14 and CD68 expression within CD11b^+^ CD45^low/int^ Ly6C^-^ microglia (**Supplementary Figure 2**).

Panel 3: IL-3 and IL-3R in astrocytes and microglia. Following exclusion of debris and doublets, CD11b and CD45 expression were used to separate myeloid (CD11b^+^ CD45^low/int^) from non-myeloid (CD11b^-^ CD45^-^) populations. Astrocytes were identified within the CD11b^-^ CD45^-^ fraction based on ACSA-2 expression, and IL-3 and GFAP expression were assessed within the ACSA-2^+^ astrocyte population. In parallel, microglia were defined as CD11b^+^ CD45^low/int^ Ly6C^-^ ACSA-2^-^ cells, and IL-3R expression was quantified within this gated microglial population (**Supplementary Figure 3**).

Panel 4: T cell and NKT cells. Following exclusion of debris, dead cells, and doublets, T cells were defined as CD3^+^ CD45^+^ cells. Within this population, NKT cells were identified as NK1.1^+^ CD3^+^ CD45^+^ cells. T cells were further subdivided into CD4^+^ CD3^+^ CD45^+^ NK1.1^-^ and CD8^+^ CD3^+^ CD45^+^ NK1.1^-^ subsets (**Supplementary Figure 4**).

Panel 5: Endothelial cell activation. Endothelial cells were identified from brain single-cell suspensions following exclusion of debris, dead cells, and doublets. Endothelial cells were defined as CD31^+^ cells, and activation status was assessed by expression of ICAM-1, VCAM-1, E-selectin (CD62E), and P-selectin (CD62P) within the CD31^+^ population (**Supplementary Figure 5**).

### 2.8 Hippocampus homogenization

Frozen tissue samples were homogenized to extract total protein. Approximately 15-25mg of tissue was placed into a 1.5mL microcentrifuge tube containing 350μL of lysis buffer (50mM Tris HCl pH 7.5, 150mM NaCl, 2mM Na2EDTA, 1% NP-40, 0.25% deoxycholate) supplemented with protease (Millipore Sigma #539134) and phosphatase (Millipore Sigma #524629) inhibitors. Four to five 0.5mm silica glass beads were then added to the samples before being mechanically homogenized using the Next Advance Bullet Blender^®^ GOLD for 4 minutes at a speed setting of 4. The silica glass beads were subsequently removed from the homogenized solution before being centrifuged at 4 °C 22,000 xg for 30 minutes. After centrifugation, sample supernatant was carefully collected and aliquoted before being frozen at -80 °C.

### 2.9 Assessment of proinflammatory cytokines

Proinflammatory cytokines in hippocampal homogenates were quantified using the V-Plex Proinflammatory Panel 1 Mouse Kit (Meso Scale Discovery #K15048D-1). Total protein concentrations of all individual samples were measured through the BCA Protein Assay (Thermo Fisher Scientific #23228) and normalized to a concentration of 2mg/mL prior to sample loading. 25μL of normalized sample was loaded in duplicate into each well, and all subsequent steps were performed according to the manufacturer’s instructions.

### 2.10 Statistical analysis

Statistical analyses were performed using GraphPad Prism (GraphPad Software, San Diego, CA, USA) and R. For most datasets, three-way analysis of variance (ANOVA) was used to evaluate the effects of genotype, age, sex, and their interactions, followed by Bonferroni *post hoc* correction for multiple comparisons where appropriate. Recognition Index, Discrimination Index, cytokine, and flow cytometry datasets were analyzed as quantitative variables. Summary outputs of three-way ANOVA analyses are provided in **Supplementary Table 3**. Because nesting behavior represents an ordinal/discrete outcome measure, additional nonparametric analyses were performed to validate the principal findings. These included Kruskal–Wallis tests followed by Dunn’s multiple-comparison analyses, with Mann–Whitney tests used where appropriate. Nonparametric analyses were performed in R, and corresponding statistical outputs are provided in **Supplementary Table 4**. Data are presented as mean ± standard deviation (SD), and statistical significance was defined as p < 0.05.

## 3 Results

### 3.1 HLA-DRB1*15:01 expression leads to age- and sex-dependent behavioral impairments

To determine whether expression of the human MS risk allele HLA-DRB1*15:01 is associated with age-dependent neurobiological dysfunction, wild-type (WT) and humanized HLA mice were assessed at 6, 9, and 15 months of age using behavioral assays that serve as functional readouts of brain integrity. These included measures of daily living, goal-directed behavior (nest building, which is known to be impaired in several neurological disorders and diseases), and cognitive function, specifically recognition memory (novel object recognition) and spatial memory (object-location discrimination) (24, 27–29).

**Figure 1:**
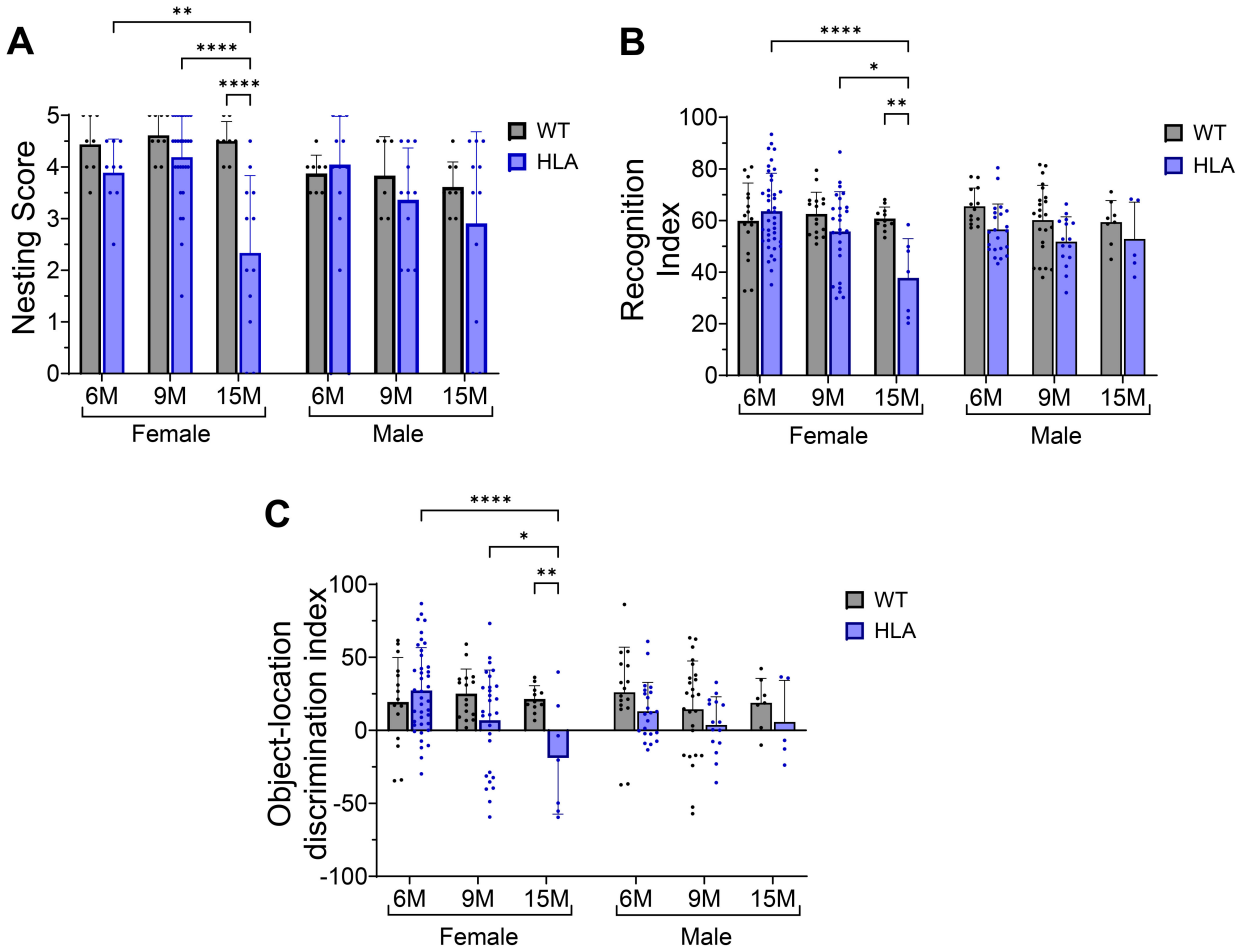
Behavioral assessment of wild-type and HLA-DRB1*15:01 mice across age and sex. Wild-type (WT, gray) and HLA-DRB1*15:01 (HLA, blue) mice were tested at 6, 9, and 15 months of age. **(A)** Nest-building score, **(B)** object-location novel object recognition (NOR) index, and **(C)** discrimination index. Bars represent mean ± SD with individual data points shown; n = 10–24 mice per group. Statistical analyses were performed using two-way or three-way ANOVA, as appropriate, followed by Bonferroni’s multiple-comparisons test. *p < 0.05, **p < 0.01, ****p < 0.0001.

Behavioral performance was preserved in WT mice of both sexes across all ages examined and in HLA male mice at all ages (**Figure 1**). In contrast, behavioral abnormalities emerged selectively in aging HLA female mice. Three-way ANOVA of nesting behavior revealed significant effects of age (p = 0.0017), genotype (p = 0.0327), and sex (p = 0.0002), as well as significant age × sex (p = 0.0131) and genotype × sex (p = 0.0493) interactions, supporting sex-dependent effects of HLA-DRB1*15:01 on behavioral performance (**Figure 1A**). Because nesting scores represent ordinal behavioral outcomes, additional nonparametric analyses were performed to validate the principal findings. Kruskal–Wallis analysis demonstrated significant differences among female groups (p = 0.000345), whereas male groups showed no significant differences (p = 0.398). *Post hoc* Dunn’s multiple-comparison analyses confirmed that 15-month-old HLA female mice exhibited significantly reduced nesting scores compared with age-matched WT female mice (adjusted p = 0.0068), as well as compared with 6-month-old HLA female mice (adjusted p = 0.029) and 9-month-old HLA female mice (adjusted p = 0.002). No significant differences were detected among WT female groups or among male groups. Collectively, these findings demonstrate that nesting impairments emerge selectively in aged HLA female mice.

Deficits in daily living behavior were accompanied by impairments in recognition memory, as assessed using the novel object recognition (NOR) task (**Figure 1B**). 15-month-old HLA female mice displayed a 40% reduction in recognition index compared with age-matched WT female mice (p = 0.0043). Recognition index values in 15-month-old HLA female mice were also significantly reduced compared with 6-month-old HLA female mice (p < 0.0001) and 9-month-old HLA female mice (p = 0.019). Analysis of object-location discrimination (**Figure 1C**) revealed a similar pattern. 15-month-old HLA female mice exhibited negative discrimination indices, indicating impaired spatial recognition. These values were significantly reduced compared with age-matched WT female mice (p= 0.0039), 6-month-old HLA female mice (p < 0.0001), and 9-month-old HLA female mice (p= 0.029).

Together, these findings identify a late-onset behavioral phenotype restricted to female HLA mice, providing functional evidence that HLA-DRB1*15:01 expression is associated with increased vulnerability of the aging female brain. This behavioral profile motivated subsequent analyses of the cellular and molecular neuroimmune mechanisms underlying this phenotype.

Female HLA mice also exhibited an age-associated transition in reproductive cycling status, with regular cycling predominating at 6 months, mixed cycling patterns observed at 9 months, and predominantly acyclic status at 15 months, consistent with reproductive aging (**Supplementary Figure 6A**). Exploratory endocrine-stage stratification analyses performed at 9 months did not reveal substantial or consistent endocrine-stage–dependent differences across the measured immune, glial, or endothelial parameters (**Supplementary Figures 6B–O**). Therefore, female mice were analyzed together in the primary dataset.

### 3.2 HLA-DRB1*15:01 expression is associated with age- and sex-dependent microglial alterations in the brain

Mitochondrial oxidative stress in microglia is an indicator of neuroimmune dysfunction and has been implicated in age-related neuroinflammatory and neurodegenerative processes (30, 31). We therefore examined whether the behavioral vulnerability observed in HLA mice was accompanied by age- and sex-dependent alterations in microglial oxidative state.

**Figure 2:**
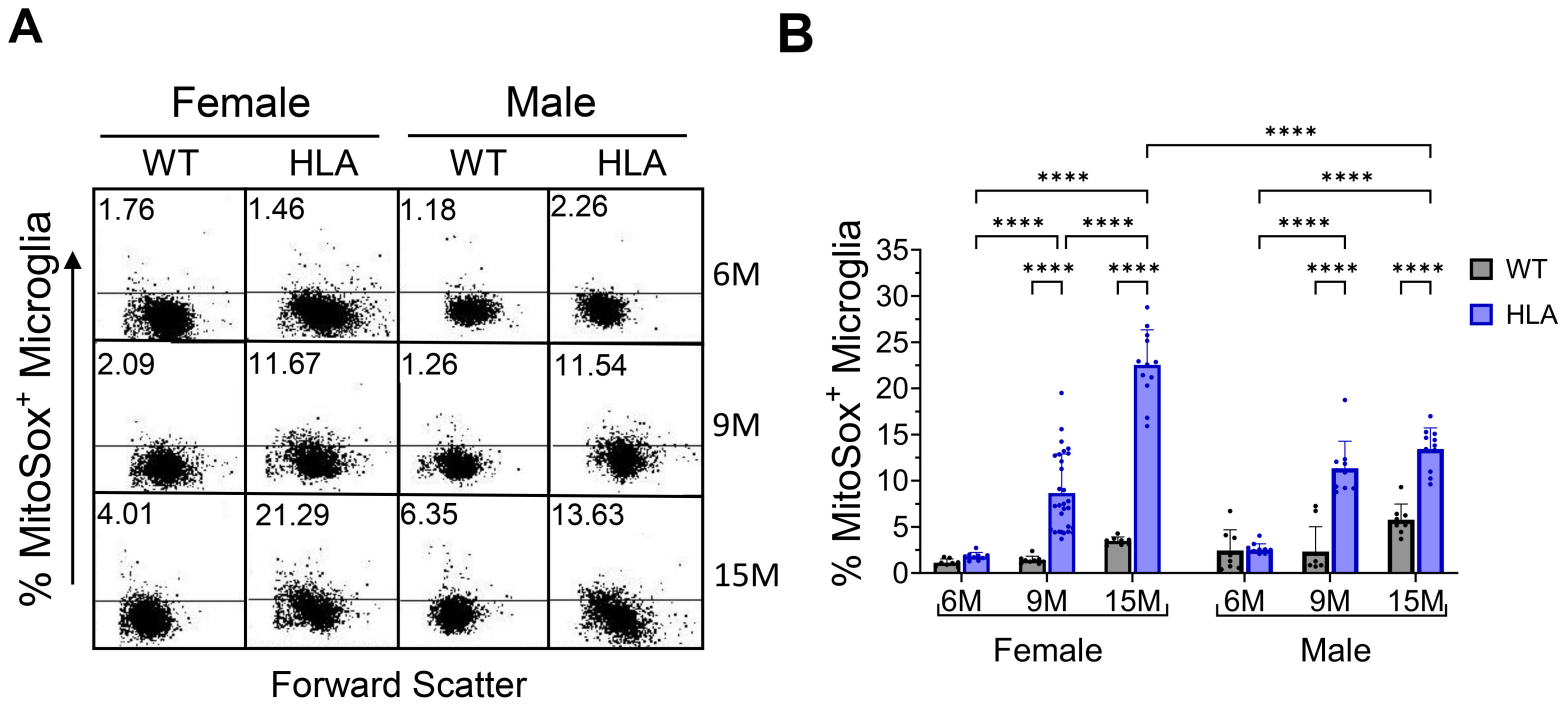
Microglial mitochondrial oxidative stress across age and sex in wild-type and HLA-DRB1*15:01 mice. Brain cells from female and male wild-type (WT, gray) and HLA-DRB1*15:01 (HLA, blue) mice at 6, 9, and 15 months of age (6M, 9M, 15M) were dissociated, stained with cell type-specific markers, and analyzed by flow cytometry. Microglia were identified as Ly6C^−^ CD11b^+^ CD45^int cells. Mitochondrial oxidative stress was evaluated using MitoSox fluorescence as a readout of mitochondrial oxidant burden, and data are expressed as the percentage of MitoSox^+^ microglia. **(A)** Representative flow cytometry dot plots. **(B)** Quantitative analysis. Bars represent mean ± SD with individual mice shown (n = 8–18 per group). Data were analyzed by three-way ANOVA (Age × Sex × Genotype) followed by Bonferroni’s multiple-comparisons test. *p < 0.05, **p < 0.01, ****p < 0.0001. Significant main effects of age and genotype, as well as significant interaction effects, were detected.

Mitochondrial oxidative stress in brain microglia was evaluated using MitoSOX fluorescence as a readout of mitochondrial oxidant burden (32). Representative flow cytometry dot plots are shown in **Figure 2a**, with quantitative analysis presented in **Figure 2b**. In HLA female mice, the proportion of MitoSOX-positive microglia increased from approximately 1.5% at 6 months to ~11.5% at 9 months, reaching ~21% at 15 months. In HLA male mice, MitoSOX-positive microglia increased from approximately 1.0% at 6 months to ~11.5% at 9 months, reaching ~13.5% at 15 months. In contrast, WT mice of both sexes displayed consistently low frequencies of MitoSOX-positive microglia across all ages examined, remaining below 5% (**Figures 2A, B**).

*Post hoc* analyses demonstrated that HLA mice of both sexes exhibited significantly higher frequencies of MitoSOX-positive microglia than age-matched WT mice at 9 and 15 months (e.g., 15-month WT male vs 15-month HLA male, p < 0.0001), whereas no significant genotype differences were detected at 6 months. Notably, at 15 months of age, the frequency of MitoSOX-positive microglia was higher in HLA females than in HLA males, indicating a sex-dependent divergence in microglial oxidative stress at advanced age (**Figure 2B**).

Together, these findings indicate that expression of HLA-DRB1*15:01 is associated with early, age- and sex-dependent alterations in microglial oxidative state, which emerge prior to overt inflammatory activation. The selective amplification of oxidative stress in aged HLA females parallels the late-onset behavioral vulnerability observed in this group, suggesting a potential cellular context in which downstream neuroimmune changes may occur. These observations prompted further examination of whether increased oxidative burden is associated with alterations in microglial activation phenotype.

### 3.3 HLA-DRB1*15:01 expression is associated with age- and sex-dependent microglial activation in the brain

Microglial activation is a central cellular feature of neuroinflammatory and neurodegenerative processes (31, 33). We examined whether the age-dependent increase in microglial oxidative stress was accompanied by changes in microglial activation state. MHC class II expression was not used as a marker of microglial activation in this study, as the HLA-DRB1*15:01 humanized mouse model lacks endogenous murine MHC class II expression. Accordingly, microglial activation was evaluated using CD14, a pattern-recognition receptor associated with innate immune and inflammatory signaling (34–36), and CD68, a lysosomal/phagolysosomal marker associated with microglial activation and phagocytic activity (37). These markers provide MHC-II–independent measures suitable for comparative analyses between WT and HLA mice.

**Figure 3:**
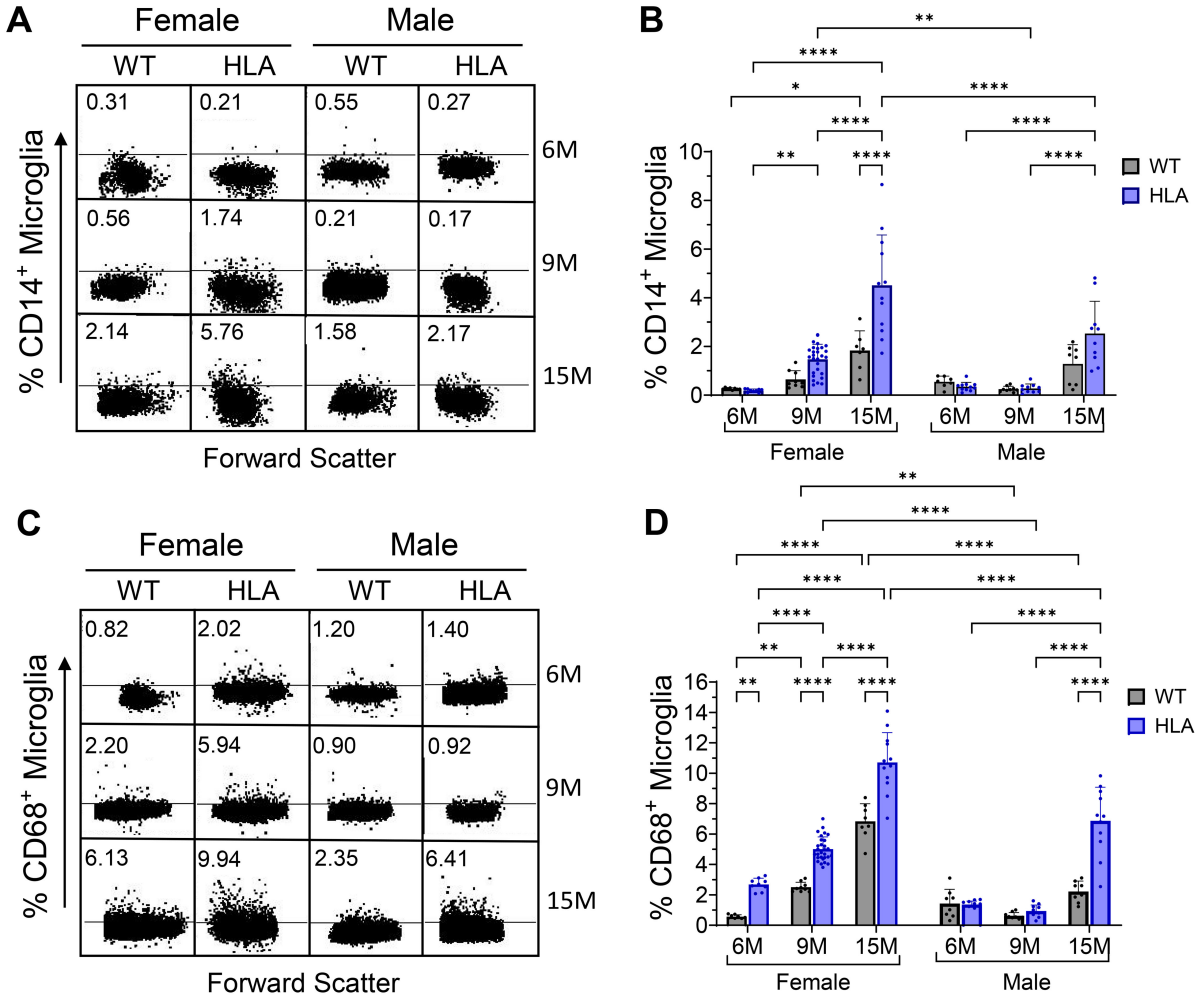
Age- and sex-dependent microglial immune-associated changes in wild-type and HLA-DRB1*15:01 mice. Brain cells were dissociated from female and male wild-type (WT, gray) and HLA-DRB1*15:01 (HLA, blue) mice at 6, 9, and 15 months of age, stained with cell type-specific markers, and analyzed by flow cytometry. Cells were gated based on forward- and side-scatter characteristics, with dead cells and doublets excluded. Microglia were identified as Ly6C^−^ CD11b^+^ CD45^int^ cells. **(A)** Representative flow cytometry dot plots and **(B)** quantification of CD14^+^ microglia, a pattern-recognition receptor associated with innate immune and inflammatory signaling. **(C)** Representative flow cytometry dot plots and **(D)** quantification of CD68^+^ microglia, a lysosomal/phagolysosomal marker associated with reactive microglial states. Bars represent mean ± SD, with individual mice shown (n = 8–18 per group). Data were analyzed by three-way ANOVA (Age × Sex × Genotype) followed by Bonferroni’s multiple-comparisons test. *p < 0.05, **p < 0.01, ****p < 0.0001. For CD14^+^ microglia, significant main effects of age (p < 0.0001), sex (p = 0.0002), and genotype (p < 0.0001) were detected, along with significant Age × Sex (p = 0.0013), Age × Genotype (p < 0.0001), and Sex × Genotype (p = 0.0157) interactions; the Age × Sex × Genotype interaction was not significant (p = 0.2797). For CD68^+^ microglia, three-way ANOVA revealed significant main effects of age, sex, and genotype (all p < 0.0001), as well as significant Age × Sex, Age × Genotype, Sex × Genotype (p = 0.0024), and Age × Sex × Genotype (p = 0.0022) interactions.

Brain cells were isolated from wild-type (WT) and HLA-DRB1*15:01 (HLA) mice at 6, 9, and 15 months of age, in both females and males, and analyzed by flow cytometry (**Figure 3**). In HLA female mice, the proportion of CD14^+^ microglia increased progressively with age, rising from <0.5% at 6 months to approximately 2% at 9 months, and exceeding 5% at 15 months. WT females also showed an age-associated increase, with significantly higher CD14^+^ microglia at 15 months compared with 6 months, but levels remained substantially lower than those observed in age-matched HLA females. *Post hoc* analyses confirmed that HLA females exhibited significantly higher frequencies of CD14^+^ microglia than WT females at 15 months (**Figures 3A, B**). In HLA male mice, CD14^+^ microglia remained below 0.5% at 6 and 9 months, but increased to approximately 2.0% at 15 months, whereas WT males showed no significant age-dependent increase. As a result, a sex difference was evident at 15 months, with higher frequencies of CD14^+^ microglia in HLA females (~5%) than in HLA males (~2%) (**Figures 3A, B**).

We next assessed CD68 expression to evaluate phagolysosomal activation of microglia. In female mice, the frequency of CD68^+^ microglia increased with age in both genotypes. WT females showed an increase from approximately 1% at 6 months to ~2% at 9 months and ~6% at 15 months, whereas HLA females exhibited a larger increase, from approximately 2% at 6 months to ~6% at 9 months and ~10% at 15 months (**Figures 3C, D**). *Post hoc* analyses demonstrated that HLA females had significantly higher frequencies of CD68^+^ microglia than age-matched WT females at 6 months (p = 0.0012), 9 months (p < 0.0001), and 15 months (p < 0.0001) (**Figure 3D**). In male mice, WT animals showed no significant age-dependent changes in CD68^+^ microglia (< 2.5%). In contrast, HLA males showed CD68 expression levels of ~1–1.5% at 6 and 9 months, but exhibited a marked increase at 15 months to ~6.5%, which was significantly higher than those of age-matched WT males (p < 0.0001) (**Figures 3C, D**). Consistent with the significant Sex × Genotype and Age × Sex × Genotype interactions, sex-dependent differences in CD68^+^ microglia were evident at both 9 and 15 months, with higher frequencies in HLA females (~6% vs ~1% at 9 months; ~10% vs ~6.5% at 15 months) compared with HLA males (**Figures 3C, D**).

These data show that HLA-DRB1*15:01 expression is associated with progressive, age- and sex-dependent microglial immune-associated changes, encompassing alterations in innate immune signaling, oxidative stress, and phagolysosomal pathways. The greater magnitude of these changes observed in aged HLA females suggests that microglial responses to aging are modulated by sex in the context of HLA expression. Given the central role of microglia in shaping the neuroimmune environment, these findings raised the possibility that intercellular glial communication may also be altered in HLA mice.

### 3.4 HLA-DRB1*15:01 promotes age- and sex-dependent astrocyte–microglia immune signaling

Astrocytes and microglia actively participate in neuroinflammation by regulating the innate immune system. Given the emergence of age- and sex-dependent behavioral impairments, increased microglial oxidative stress, and changes in activation-associated microglial markers in mice expressing HLA-DRB1*15:01, we next examined whether these phenotypes were accompanied by alterations in glial immune signaling pathways that regulate microglial activation state. Because astrocyte–microglia communication plays a central role in shaping neuroinflammatory responses, we focused on the IL-3/IL-3 receptor axis, which rather than serving as a direct marker of activation. We therefore quantified age-, sex-, and genotype-dependent changes in IL-3R expression on microglia, IL-3 production by astrocytes, and astrocyte reactivity to assess coordinated glial immune signaling in WT and HLA mice.

**Figure 4:**
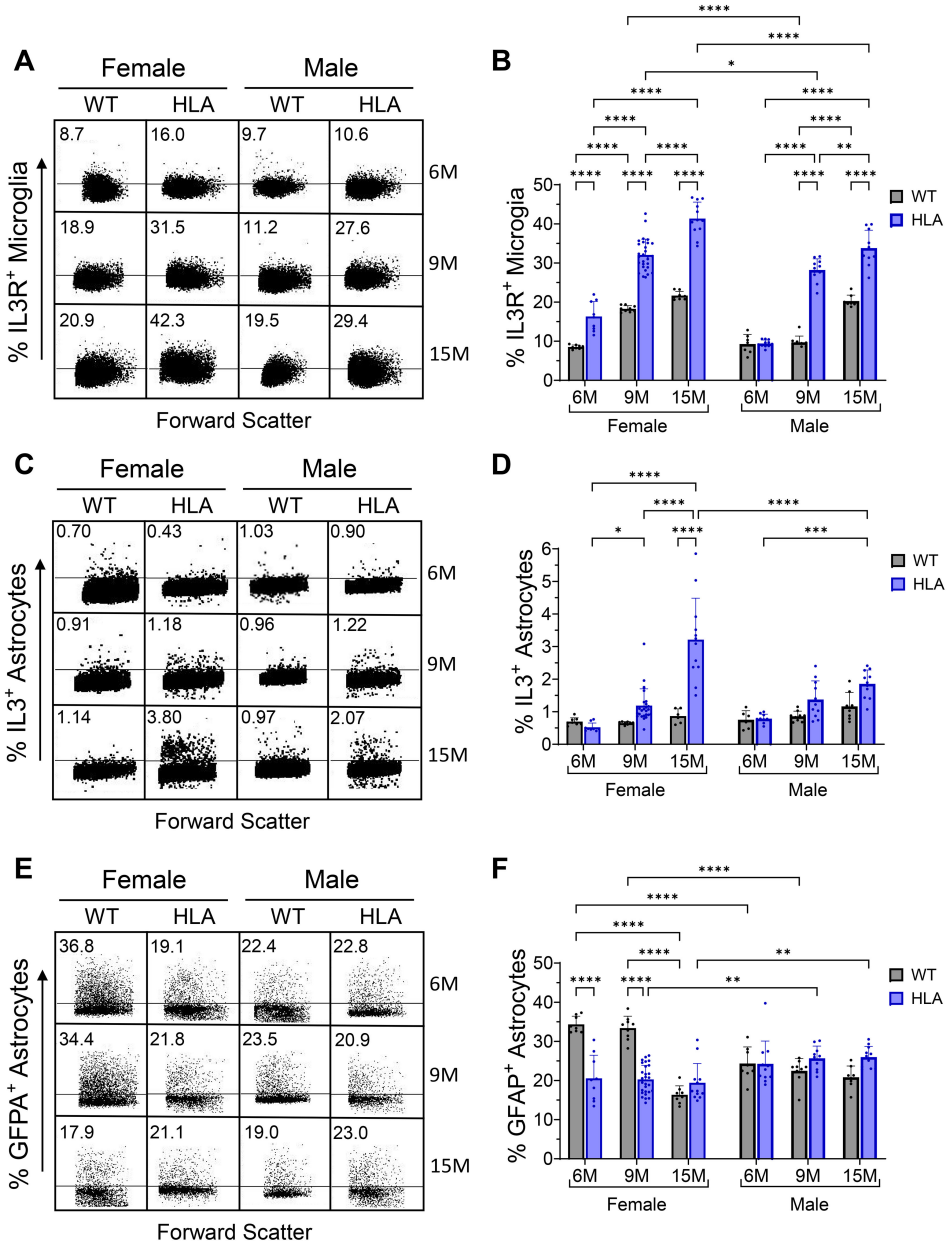
Age- and sex-dependent modulation of astrocyte–microglia IL-3/IL-3R signaling by HLA-DRB1*15:01. Brain cells were dissociated from female and male wild-type (WT, gray) and HLA-DRB1*15:01 (HLA, blue) mice at 6, 9, and 15 months of age, stained with cell type-specific markers, and analyzed by flow cytometry. Cells were gated based on forward- and side-scatter characteristics, with dead cells and doublets excluded using DAPI staining and FSC-A/FSC-H parameters. Microglia were identified as Ly6C− CD11b^high^ CD45^int^ cells, and astrocytes were identified by ACSA-2 expression. **(A)** Representative flow cytometry dot plots and **(B)** quantification of IL-3R^+^ microglia. **(C)** Representative flow cytometry dot plots and **(D)** quantification of IL-3^+^ astrocytes. **(E)** Representative flow cytometry dot plots and **(F)** quantification of GFAP^+^ astrocytes. Bars represent mean ± SD, with individual mice shown (n = 8–18 per group). Data were analyzed by three-way ANOVA (Age × Sex × Genotype) followed by Bonferroni’s multiple-comparisons test. *p < 0.05, **p < 0.01, ****p < 0.0001. Significant main effects of age and genotype, as well as significant interaction effects, were detected for IL-3R^+^ microglia, IL-3^+^ astrocytes, and GFAP^+^ astrocytes, indicating age-, sex-, and genotype-dependent modulation of astrocyte–microglia IL-3/IL-3R signaling.

IL-3R^+^ microglia increased robustly with age in both genotypes, with consistently higher frequencies in females than in males (**Figures 4A, B**). In WT females, IL-3R^+^ microglia increased from ~9% at 6 months to ~19% at 9 months and ~21% at 15 months, whereas HLA females showed a markedly greater increase, rising from ~16% at 6 months to ~32% at 9 months and ~42% at 15 months. HLA females exhibited significantly higher IL-3R^+^ microglial frequencies than age-matched WT females at all ages examined (all p < 0.0001). In males, WT mice showed relatively stable IL-3R^+^ microglial frequencies (~10%) at 6 and 9 months, followed by an increase to ~20% at 15 months. HLA males exhibited a significant age-dependent increase, rising from ~11% at 6 months to ~28% at 9 months and ~33% at 15 months, with levels at 15 months significantly higher than those of age-matched WT males (p < 0.0001) (**Figure 4B**). Across both genotypes, females exhibited higher IL-3R^+^ microglial frequencies than males, indicating a sex bias that became more pronounced with aging.

To determine whether elevated microglial IL-3R expression was accompanied by parallel regulation of astrocytic cytokine production, IL-3 expression in astrocytes was assessed (**Figures 4C, D**). In female HLA mice, IL-3^+^ astrocytes increased significantly with age, rising from ~0.5% at 6 months to ~1% at 9 months and reaching ~4% at 15 months, a response significantly greater than that observed in WT females or HLA males. Male HLA mice exhibited a more modest increase, from ~1% at 6 months to ~2% at 15 months. In contrast, WT mice of both sexes showed no age-dependent change in IL-3^+^ astrocyte frequency. These findings indicate selective, age-dependent amplification of astrocytic IL-3 expression in HLA-DRB1*15:01 mice, with a significantly stronger effect in females (**Figure 4D**).

Astrocyte reactivity was assessed by quantifying GFAP^+^ astrocytes (**Figures 4E, F**). In WT females, GFAP^+^ astrocyte frequencies were highest at 6 and 9 months (~30–35%) and declined significantly by 15 months (~18%). In contrast, female HLA mice did not exhibit a comparable age-associated decline and showed significantly lower GFAP^+^ astrocyte frequencies than age-matched WT females at 6 and 9 months. In males, GFAP^+^ astrocyte frequencies remained relatively stable across age and genotype (~20–25%). As a result, marked sex differences were evident in WT mice at younger ages (6 and 9 months), with females exhibiting higher GFAP^+^ astrocyte levels than males, whereas in HLA mice, females exhibited lower GFAP^+^ astrocyte levels than males at older ages (9 and 15 months) (**Figures 4E, F**). These patterns indicate genotype-dependent modulation of astrocyte reactivity across the lifespan.

**Figure 5:**
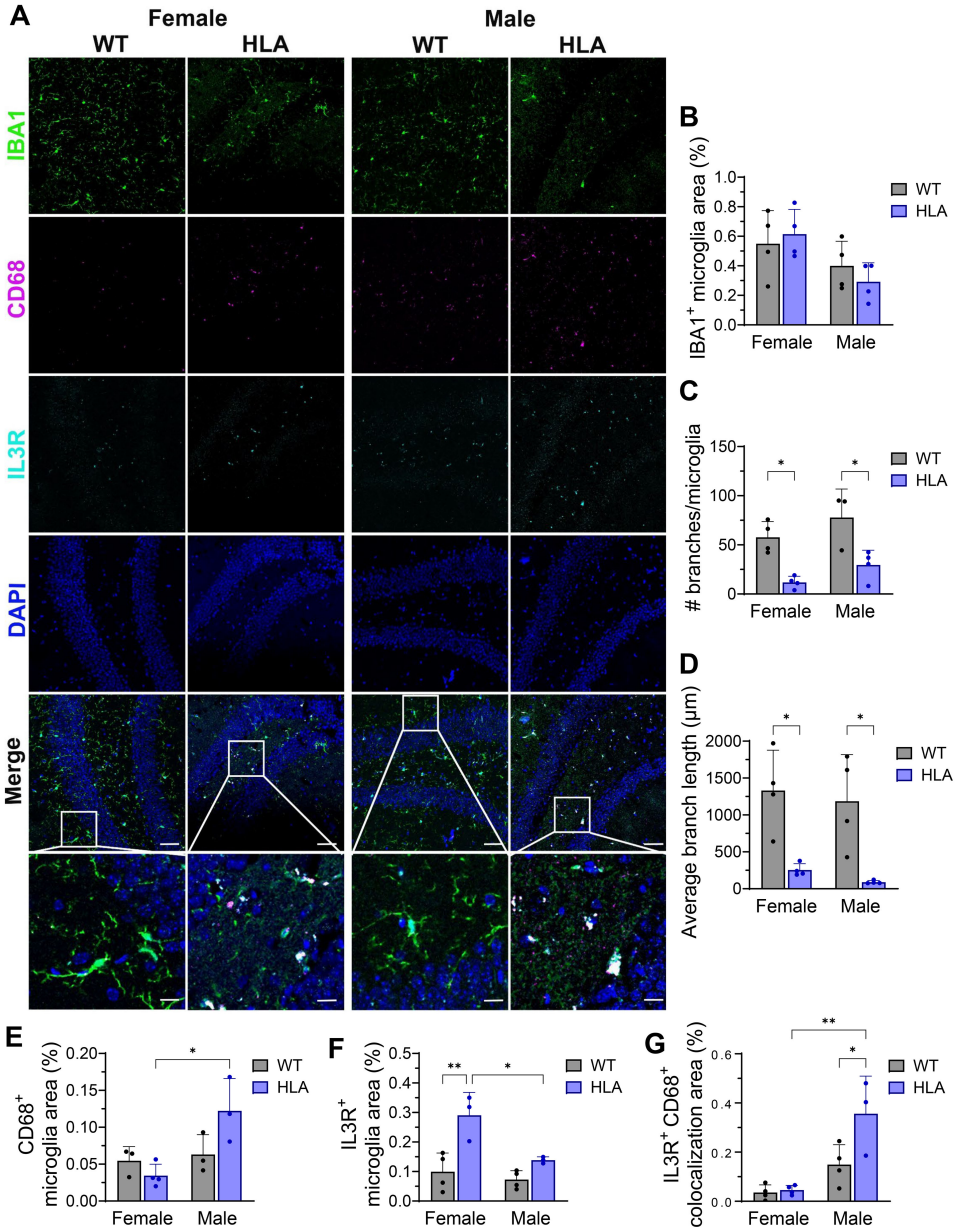
Hippocampal microglial IL-3R and CD68 immunofluorescence in aged wild-type and HLA-DRB1*15:01 mice. **(A)** Representative immunofluorescence images of hippocampal sections from 15-month-old female and male wild-type (WT) and HLA-DRB1*15:01 (HLA) mice. Sections were stained for IBA1 (microglia, green), the lysosomal/phagolysosomal marker CD68 (magenta), and IL-3 receptor (IL-3R, cyan), with nuclei counterstained with DAPI (blue). Columns correspond to female WT, female HLA, male WT, and male HLA mice. Images are shown as single-channel and merged views to illustrate cellular localization and marker distribution, with enlarged dentate gyrus regions shown in the inset panels. **(B)** Quantification of IBA1^+^ microglial immunoreactive area. **(C)** Quantification of microglial branch number and **(D)** branch length measured using Fiji (ImageJ). **(E)** Quantification of CD68^+^ microglial immunoreactive area, **(F)** IL-3R^+^ microglial immunoreactive area, and **(G)** IL-3R^+^/CD68^+^ colocalization measured using QuPath. Scale bars: 100 µm; inset panels, 25 µm. Error bars represent mean ± SEM. Statistical analyses were performed using two-way ANOVA followed by Bonferroni multiple-comparison testing. *p < 0.05, **p < 0.01, ***p < 0.001, ****p < 0.0001.

To determine whether microglial IL-3R expression and activation-associated markers were evident within intact hippocampal tissue at advanced age, we performed immunofluorescence analysis in 15-month-old WT and HLA-DRB1*15:01 mice (**Figure 5**). Representative images of IBA1, CD68, IL-3R, DAPI, and merged staining in female and male WT and HLA mice are shown in **Figure 5A**. Across hippocampal fields, IBA1^+^ microglia in WT mice exhibited a more ramified morphology with elongated and branched processes, whereas HLA mice displayed microglial profiles with reduced process complexity and shorter processes, particularly evident in the higher-magnification inset images (**Figure 5A**). Quantification of total IBA1^+^ microglial area showed relatively modest differences between WT and HLA mice (**Figure 5B**), suggesting that the observed phenotype was not primarily driven by major changes in overall microglial abundance. In contrast, morphometric analysis demonstrated reduced branching complexity and shorter branch length in HLA mice compared with WT controls (**Figures 5C, D**), consistent with altered microglial morphology. These reductions were observed in both sexes.

Quantification of activation- and signaling-associated markers further demonstrated sex-dependent alterations in microglial immune signaling. CD68^+^ microglial area demonstrated sex-dependent differences within HLA mice, with higher CD68 immunoreactivity observed in HLA males compared with HLA females (**Figure 5E**). In contrast, IL-3R^+^ microglial area was significantly increased in HLA females compared with WT females and HLA males (**Figure 5F**). IL-3R^+^CD68^+^ colocalization area was also elevated in HLA males relative to HLA females and WT males (**Figure 5G**), indicating sex-dependent differences in activation-associated IL-3R signaling. Merged images demonstrated that IL-3R and CD68 signals were present within IBA1^+^ microglial fields in HLA mice, IL-3R^+^CD68^+^ colocalization area was minimal in females of both genotypes but significantly increased in HLA males compared with WT males and HLA females (**Figure 5G**), indicating enhanced activation-associated IL-3R signaling specifically in male HLA microglia.

Together, these findings indicate that aging HLA-DRB1*15:01 mice exhibit altered astrocyte–microglia immune signaling characterized by progressive increases in IL-3R^+^ microglia, enhanced IL-3-associated signaling, and changes in activation-associated microglial phenotype. Whole-brain flow cytometry demonstrated the strongest IL-3R^+^ microglial and IL-3^+^ astrocyte responses in HLA females. Complementary hippocampal immunofluorescence analysis at 15 months provided tissue-level evidence of altered microglial phenotype in HLA mice, including reduced process complexity and shorter branch length, together with sex-dependent patterns of CD68- and IL-3R-associated signaling. Specifically, IL-3R^+^ microglial area was highest in HLA females, whereas CD68^+^ microglial area and IL-3R^+^CD68^+^ colocalization were most evident in HLA males. Thus, although whole-brain flow cytometry and hippocampal immunofluorescence identified partially distinct sex-dependent patterns, both approaches consistently support altered IL-3/IL-3R-associated glial immune signaling and microglial remodeling in aged HLA-DRB1*15:01 mice.

### 3.5 HLA-DRB1*15:01 expression is associated with age- and sex-dependent alterations in hippocampal microglial, neuronal, and myelin organization

Immune signaling in the brain occurs within a highly organized cellular environment in which the spatial relationships among glial cells, neurons, and myelin influence neuroimmune interactions. Sustained or dysregulated immune signaling can remodel tissue architecture, and we therefore examined whether the age- and genotype-dependent microglial and astrocytic phenotypes identified earlier were accompanied by corresponding changes in hippocampal cellular organization. Using immunofluorescence in 15-month-old WT and HLA mice, we assessed the spatial relationships among microglia, axonal structure, myelin, and astrocytic markers (GFAP and IL-3) within the hippocampus.

**Figure 6:**
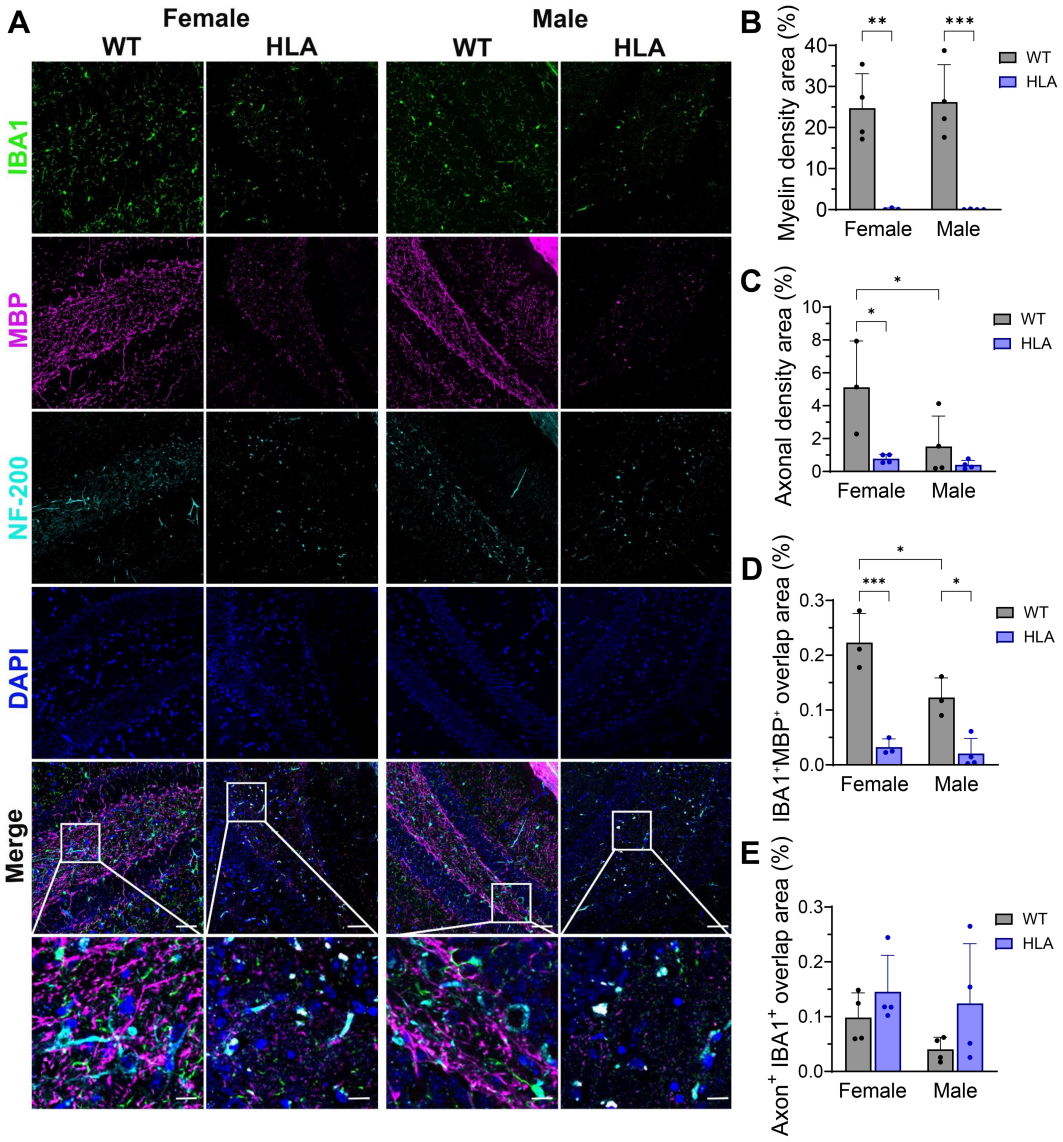
Tissue-level organization of microglia, myelin, and axonal structures in the aged hippocampus of wild-type and HLA-DRB1*15:01 mice. Hippocampal sections from 15-month-old female and male wild-type (WT) and HLA-DRB1*15:01 (HLA) mice were stained for microglia (IBA1, green), axonal structures labeled by NF-200 (cyan), myelin basic protein (MBP, magenta), and nuclei (DAPI, blue). **(A)** Representative immunofluorescence images illustrating the spatial relationships among microglia, axonal structures, and myelinated regions in the hippocampal dentate gyrus. Merged images are shown together with enlarged inset regions. **(B)** Quantification of myelin density area, **(C)** axonal density area, **(D)** IBA1^+^/MBP^+^ overlap area, and **(E)** axon^+^/IBA1^+^ overlap area measured using QuPath. Scale bars: 100 µm; inset panels, 25 µm. Error bars represent mean ± SEM. *p < 0.05, **p < 0.01, ***p < 0.001, ****p < 0.0001 by two-way ANOVA.

As shown in **Figure 6A**, and consistent with **Figure 5**, WT mice of both sexes displayed more ramified IBA1^+^ microglia, whereas HLA mice exhibited reduced process complexity and less ramified microglial morphology. Myelin-associated MBP immunoreactivity also demonstrated marked genotype-associated differences. WT mice of both sexes exhibited dense and organized MBP labeling, whereas HLA mice showed a profound reduction in MBP-associated myelin density area (**Figure 6B**). Representative images further demonstrated altered tissue organization in HLA mice, characterized by reduced MBP signal intensity and less organized myelin-associated structure within hippocampal regions (**Figure 6A**). NF200 immunostaining revealed genotype- and sex-dependent differences in axonal-associated organization. WT females displayed the highest NF200-associated axonal density, whereas HLA females showed significantly reduced axonal density area (**Figure 6C**). Male mice exhibited lower overall NF200-associated signal across genotypes, with HLA males showing a similar trend toward reduced axonal density relative to WT males (**Figure 6C**). Quantification of spatial overlap measurements demonstrated additional alterations in microglia-associated tissue organization. IBA1^+^MBP^+^ overlap area was significantly reduced in HLA mice of both sexes relative to WT controls, with WT females exhibiting the highest overlap levels overall (**Figure 6D**). In contrast, the IBA1^+^NF200^+^ overlap area showed a trend toward higher values in HLA mice than in WT controls in both sexes, although these differences did not reach statistical significance (**Figure 6E**).

**Figure 7:**
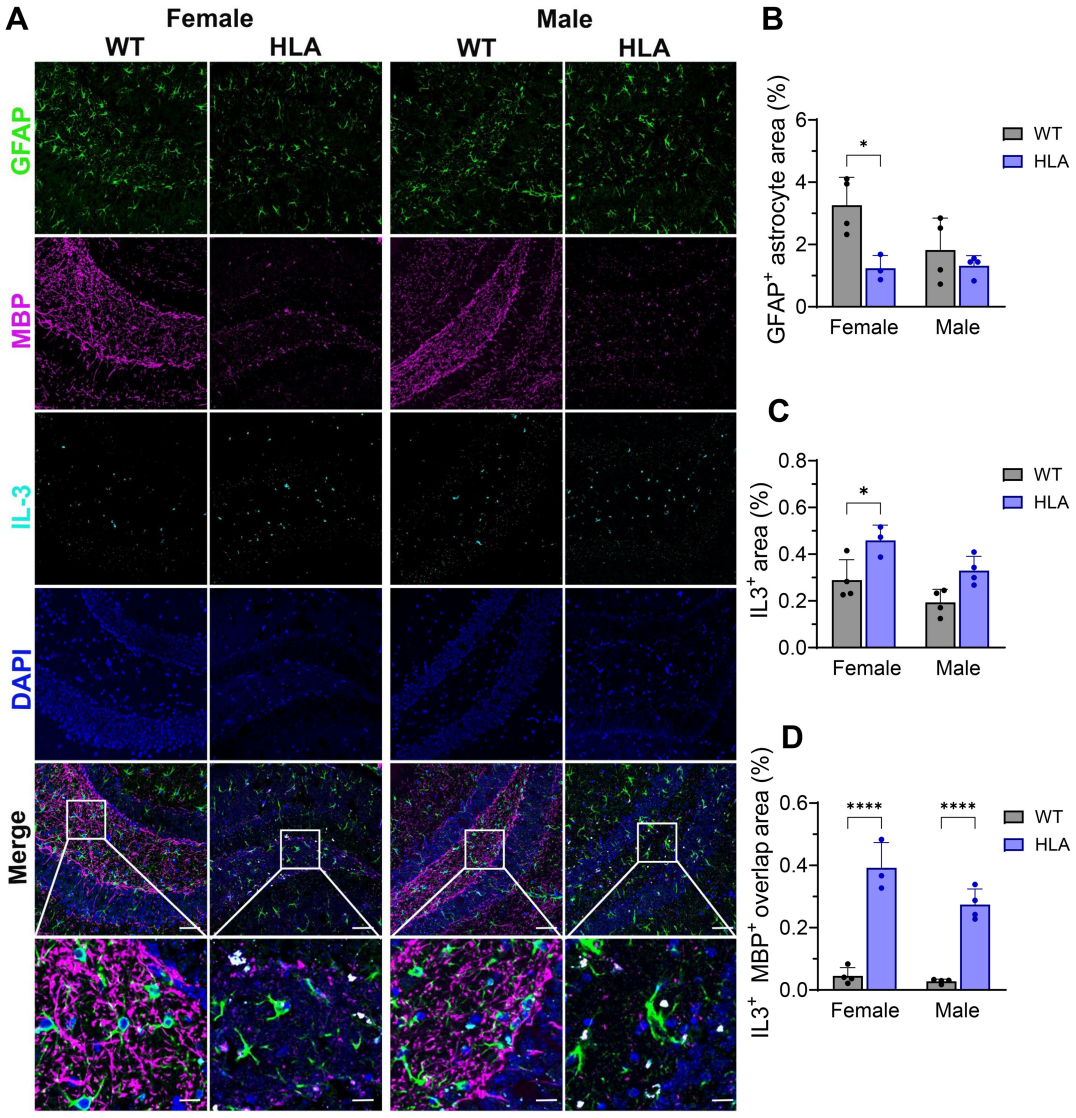
Spatial organization of astrocytic IL-3 signaling in the aged hippocampus of wild-type and HLA-DRB1*15:01 mice. Hippocampal sections from 15-month-old female and male wild-type (WT) and HLA-DRB1*15:01 (HLA) mice were stained for astrocytes labeled by GFAP (green), IL-3 immunoreactivity (cyan), myelin basic protein (MBP, magenta), and nuclei (DAPI, blue). **(A)** Representative immunofluorescence images illustrating the spatial distribution of GFAP^+^ astrocytes, IL-3 immunoreactivity, and myelinated regions within the hippocampal dentate gyrus. Images are shown as single-channel and merged views together with enlarged inset regions. **(B)** Quantification of GFAP^+^ astrocyte immunoreactive area, **(C)** total IL-3^+^ immunoreactive area, and **(D)** IL-3^+^/MBP^+^ overlap area measured using QuPath. Scale bars: 100 µm; inset panels, 25 µm. Error bars represent mean ± SEM. Statistical analyses were performed using two-way ANOVA followed by Bonferroni multiple-comparison testing. *p < 0.05, **p < 0.01, ***p < 0.001, ****p < 0.0001.

GFAP immunoreactivity in WT and HLA mice of both sexes showed widespread astrocytic labeling throughout the hippocampal field, with no evidence of marked genotype-associated increases in overall GFAP signal (**Figure 7A**). In contrast, IL-3 immunoreactivity was increased in HLA females compared with WT females (**Figure 7C**), consistent with the 15-month flow cytometry findings (**Figure 4D****).** HLA males exhibited a similar but less pronounced, non-significant increase in IL-3-associated signal relative to WT males. Across all groups, merged images revealed no detectable overlap between GFAP and IL-3 immunoreactivity, consistent with the absence of GFAP^+^IL-3^+^ colocalization observed by flow cytometry (data not shown). Although direct GFAP–IL-3 overlap was not observed, IL-3 immunoreactivity in HLA mice was frequently detected within regions exhibiting disrupted MBP-associated myelin organization. Quantification of IL-3^+^MBP^+^ overlap area demonstrated markedly increased spatial association between IL-3-associated signal and myelin-associated regions in HLA mice of both sexes compared with WT controls (**Figure 7D**). Together, these findings indicate that increased IL-3-associated signaling in aged HLA mice is associated with altered myelin organization rather than generalized astrocyte expansion.

Together, these findings indicate that aging HLA-DRB1*15:01 mice exhibit altered hippocampal tissue organization characterized by reduced microglial ramification, marked loss of MBP-associated myelin organization, changes in NF200-associated axonal structure, and altered spatial relationships among microglia, IL-3-associated signal, and myelin-associated regions. The absence of detectable GFAP–IL-3 overlap, together with the increased association between IL-3 immunoreactivity and disrupted myelin regions, suggests that altered IL-3-associated immune signaling in HLA mice occurs in parallel with structural remodeling of the hippocampal microenvironment rather than generalized astrocyte expansion.

### 3.6 HLA-DRB1*15:01 drives age- and sex-dependent immune cell recruitment and endothelial activation at CNS borders

Neuroinflammatory processes in the aging brain are shaped not only by resident glial responses but also by tightly regulated immune cell trafficking across CNS interfaces and by endothelial activation that governs leukocyte entry. Recent work has identified astrocyte-derived interleukin-3 (IL-3) as a key amplifier of neuroimmune crosstalk. Early IL-3 production by activated astrocytes can stimulate IL-3 receptor–expressing microglia to produce chemokines that promote the recruitment of CD4^+^ T cells into the CNS. Given the age- and sex-dependent alterations in microglial activation and astrocyte–microglia IL-3/IL-3R signaling observed in HLA-DRB1*15:01 mice, we next examined whether these changes were accompanied by selective lymphoid immune cell accumulation at CNS borders and within the brain parenchyma, as well as by endothelial activation at the neurovascular interface. To address this, we quantified CD3^+^ T cells, CD4^+^ T-cell subsets, and NK1.1^+^CD3^+^ natural killer T (NKT) cells in the meninges and brain across age, sex, and genotype, and assessed endothelial adhesion molecule expression associated with leukocyte recruitment. CD8^+^ T cells were not detected at appreciable levels in either compartment and were therefore excluded from further analysis.

**Figure 8:**
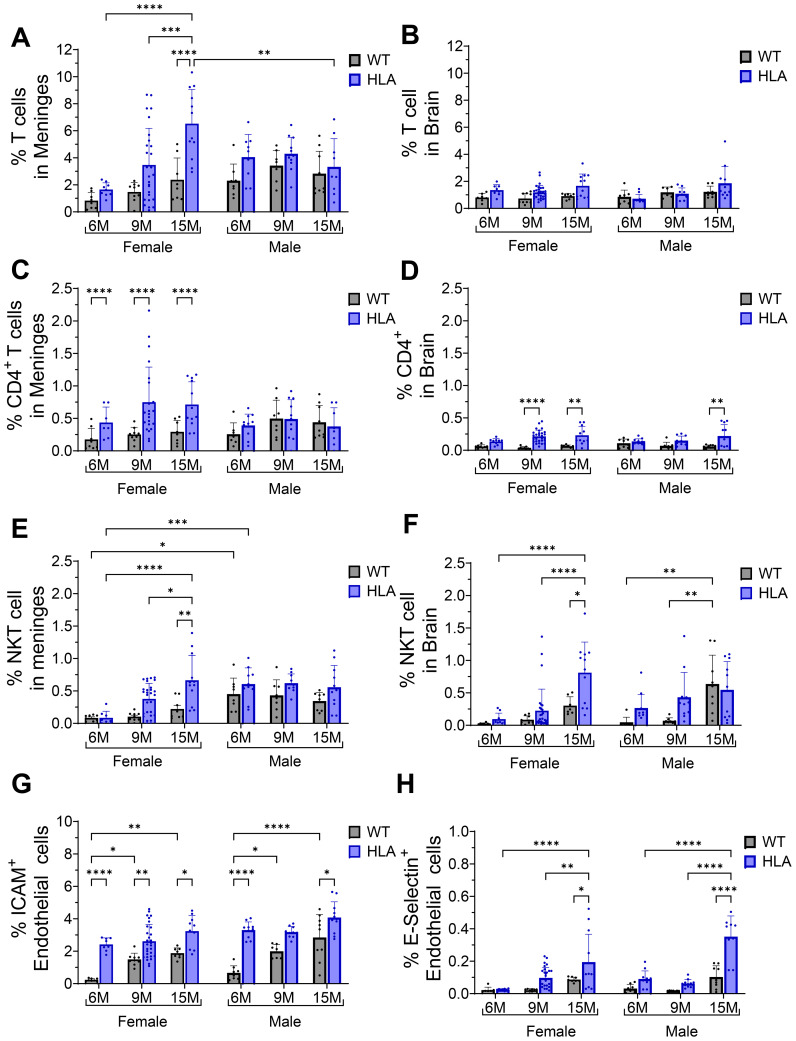
Age- and sex-dependent T cell accumulation, NKT cell responses, and endothelial activation in the meninges and brain of HLA-DRB1*15:01 mice. Meninges and brain tissue were dissociated from female and male wild-type (WT, gray) and HLA-DRB1*15:01 (HLA, blue) mice at 6, 9, and 15 months of age, stained with cell type-specific markers, and analyzed by flow cytometry. Cells were gated based on forward- and side-scatter characteristics, with dead cells and doublets excluded using DAPI staining and FSC-A/FSC-H parameters. T cells were identified as CD45^+^CD3^+^ cells, and CD4^+^ T cells were defined as CD45^+^CD3^+^CD4^+^CD8− cells. **(A)** Percentage of total T cells in the meninges and **(B)** percentage of total T cells in the brain. **(C)** Percentage of CD4^+^ T cells in the meninges and **(D)** percentage of CD4^+^ T cells in the brain. NKT cells were identified as CD45^+^CD3^+^NK1.1^+^ cells. **(E)** Percentage of NKT cells in the meninges and **(F)** percentage of NKT cells in the brain. Endothelial cells were identified as CD31^+^ cells, and endothelial activation was assessed by adhesion molecule expression. **(G)** Percentage of ICAM-1^+^ (CD54^+^) endothelial cells and **(H)** percentage of E-selectin^+^ (CD62E^+^) endothelial cells in the brain. Data are presented as mean ± SEM with individual mice shown. Statistical significance was determined by three-way ANOVA followed by Bonferroni’s multiple-comparisons test (*p < 0.05, **p < 0.01, ***p < 0.001, ***p < 0.0001). Significant main effects and interaction effects indicated age-, sex-, and genotype-dependent modulation of meningeal and brain T cell accumulation, NKT cell responses, and endothelial activation markers in HLA-DRB115:01 mice.

We quantified the percentage of total T cells in the meninges and brain across age, sex, and genotype (**Figures 8A, B**). In female mice, 15-month-old HLA animals showed a significant increase in meningeal T cell frequency (~6.5%) compared with 6-month-old HLA females (~1.5%; p < 0.0001), 9-month-old HLA females (~3.5%; p = 0.0004), and age-matched WT females (WT ~2% vs HLA ~6.5%; p < 0.0001) (**Figure 8A**). Comparison between sexes revealed a significant sex-dependent effect at 15 months, with HLA females exhibiting higher meningeal T cell frequencies than HLA males (female ~6.5% vs male ~3.5%; p = 0.004). No significant differences were detected among the remaining age, sex, or genotype groups in male and female WT mice or in male HLA mice (**Figure 8A**). In contrast, analysis of brain-infiltrating T cells revealed no statistically significant differences across age, sex, or genotype after correction for multiple comparisons (**Figure 8B**). Exploratory *post hoc* (Fisher’s LSD) analyses identified pairwise differences in brain T cell percentages in 15-month-old HLA mice of both sexes compared with WT controls (female: HLA ~1.7% vs WT ~0.9%, p = 0.0087; male: HLA ~1.8% vs WT ~1.2%, p = 0.02); however, these differences did not remain significant following correction (Bonferroni/Sidak) and should therefore be interpreted as exploratory.

Female HLA mice also exhibited a significant increase in the percentage of meningeal CD4^+^ T cells compared with age-matched WT controls at 6, 9, and 15 months (all p < 0.0001) (**Figure 8C**). However, no age-dependent increase was observed within the HLA female group. In contrast, neither WT nor HLA male mice showed significant differences in meningeal CD4^+^ T cell frequencies across age or genotype (**Figure 8C**). Analysis of CD4^+^ T cells in the brain also revealed significant genotype-dependent differences (**Figure 8D**). Female HLA animals showed a significant increase in brain CD4^+^ T cell frequency at 9 and 15 months compared with age-matched WT controls (p < 0.01 and p < 0.0001, respectively) (**Figure 8D**). HLA males showed a significant increase in brain CD4^+^ T cell frequency at 15 months relative to WT males (p < 0.01), whereas no significant differences were detected at earlier ages (**Figure 8D**).

Analysis of NK1.1^+^CD3^+^ natural killer T (NKT) cells in the meninges revealed significant age-, sex-, and genotype-dependent differences (**Figure 8E**). Compared with age-matched WT controls, HLA females exhibited significantly higher percentages of meningeal NKT cells at 15 months (p = 0.014). Within the HLA female group, NKT cell levels at 15 months were significantly elevated relative to both 6 months (p < 0.0001) and 9 months (p = 0.012) (**Figure 8E**). In contrast, HLA male mice did not exhibit significant age- or genotype-dependent differences in meningeal NKT cell frequencies. A significant sex difference was observed at 6 months in both genotypes, with males exhibiting higher percentages of meningeal NKT cells than females in both the WT (p = 0.04) and HLA (p = 0.0002) groups (**Figure 8E**). In the brain parenchyma, NKT cell analysis did not reveal a uniform sex-independent effect across genotypes (**Figure 8F**). However, similar to the meninges, brain NKT cells showed significant age- and genotype-dependent effects in females but not in males. In HLA females, NKT cell levels at 15 months were significantly higher than at both 6 months (p < 0.0001) and 9 months (p < 0.0001), and were also elevated relative to age-matched WT controls (p = 0.016) (**Figure 8F**). In contrast, HLA males did not exhibit a significant age-dependent effect. Notably, WT males showed increased brain NKT cell levels at 15 months compared with both 6 months (p = 0.002) and 9 months (p = 0.0052) (**Figure 8F**).

Endothelial activation in the brain was assessed by analyzing the expression of adhesion molecules, including P-selectin, E-selectin, VCAM-1, and ICAM-1. P-selectin and VCAM-1 were not detected at appreciable levels; therefore, subsequent analyses focused on ICAM-1 and E-selectin as indicators of endothelial activation at the neurovascular interface (**Figures 8G, H**). Analysis of ICAM-1–positive endothelial cells revealed significant age- and genotype-dependent effects (**Figure 8G**). HLA females and males did not exhibit significant age- or sex-dependent differences in ICAM-1^+^ endothelial cell percentages within the HLA genotype. However, ICAM-1 expression was consistently higher in HLA mice compared with age-matched WT controls in both sexes. In females, ICAM-1^+^ endothelial cells were significantly increased in HLA mice at 6 months (~2.5% vs ~0.3%; p < 0.0001), 9 months (~2.5% vs ~1.5%; p = 0.008), and 15 months (~3.0% vs ~2.0%; p = 0.05). Similarly, in males, HLA mice showed significantly higher ICAM-1 expression at 6 months (~3.0% vs ~0.5%; p < 0.0001) and 15 months (~4.0% vs ~3.0%; p < 0.0001) compared with WT controls. In contrast, WT females and males showed an age-associated increase in ICAM-1 expression, with significantly higher levels at 15 months than at 6 months in both sexes. E-selectin–positive endothelial cells were detected at much lower frequencies than ICAM-1–positive cells (**Figures 8G, H**). Nevertheless, E-selectin analysis revealed significant age- and genotype-dependent differences (**Figure 8H**). In female mice, HLA animals exhibited a significant increase in E-selectin^+^ endothelial cells at 15 months compared with 6 months (p < 0.0001), 9 months (p = 0.0059), and age-matched WT females (p < 0.001). In male mice, E-selectin^+^ endothelial cells were similarly increased in 15-month-old HLA animals compared with 6 months (p < 0.0001), 9 months (p < 0.0001), and age-matched WT males (p < 0.0001). No significant differences were detected at earlier ages.

Together, these findings demonstrate that HLA-DRB1*15:01 expression promotes selective, age- and sex-dependent immune remodeling at CNS interfaces, rather than generalized immune infiltration. Increased accumulation of CD4^+^ T cells and NKT cells—particularly in females—occurs alongside endothelial activation characterized by elevated ICAM-1 and E-selectin expression, suggesting a permissive neurovascular environment for regulated immune cell recruitment. When considered in the context of the preceding results showing microglial immune-associated changes, IL-3/IL-3R signaling, and tissue-level remodeling, these data support a model in which HLA-DRB1*15:01 amplifies chronic, compartmentalized neuroinflammatory processes that emerge with aging and are strongly modulated by sex.

### 3.7 HLA-DRB1*15:01 expression alters selective brain cytokine signaling with aging

**Figure 9:**
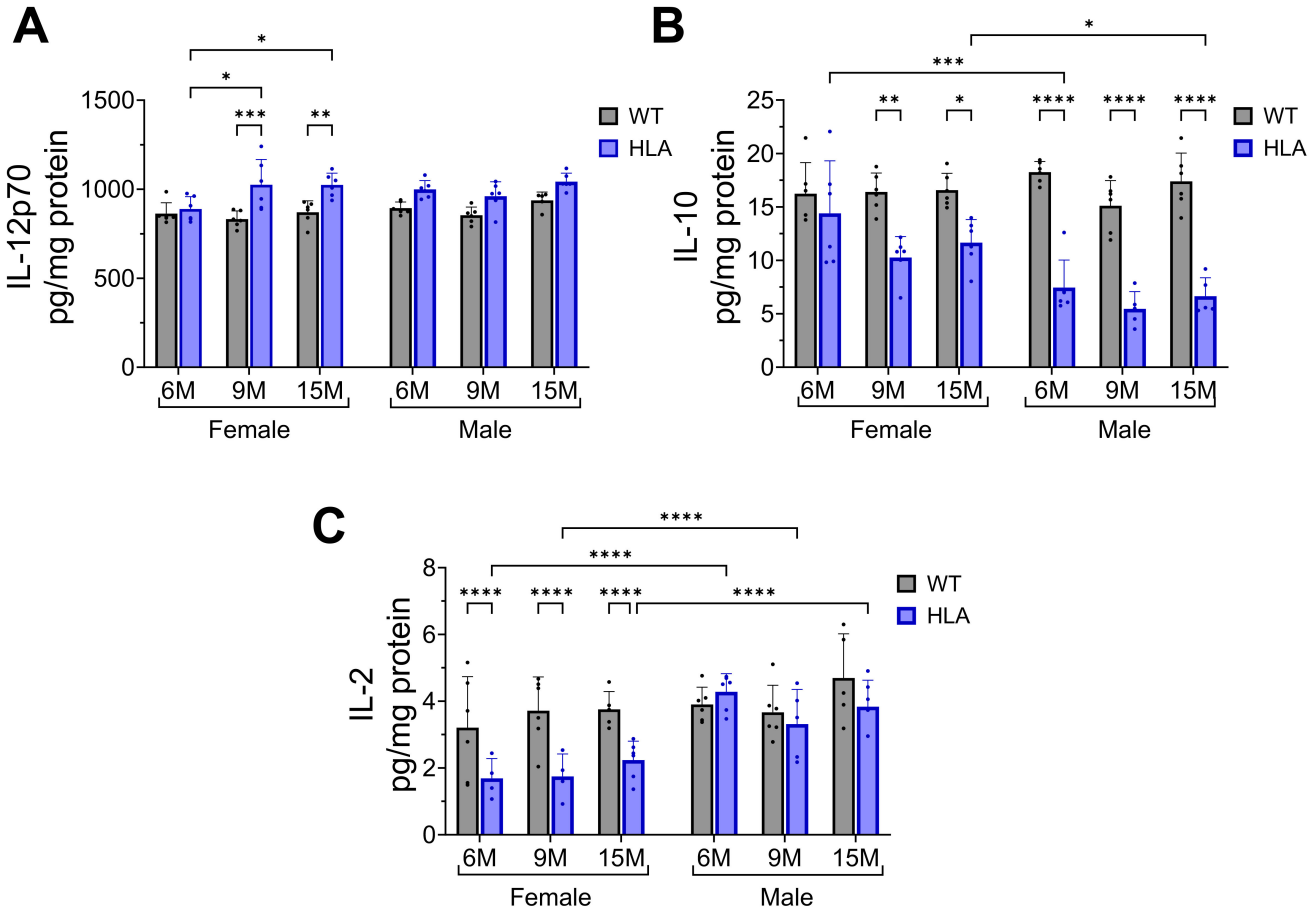
Selective alterations in hippocampal cytokine profiles with aging in HLA-DRB1*15:01 mice. Hippocampal tissue was collected from female and male wild-type (WT, gray) and HLA-DRB1*15:01 (HLA, blue) mice at 6, 9, and 15 months of age. Tissue was lysed, and cytokine concentrations were quantified using a multiplex electrochemiluminescence assay (MSD). Among the cytokines assessed, only IL-12p70, IL-10, and IL-2 were detected at levels suitable for statistical analysis. **(A)** IL-12p70, **(B)** IL-10, and **(C)** IL-2 concentrations are shown and expressed as pg/mg total protein. Data are presented as mean ± SEM with individual mice shown. Statistical significance was determined by three-way ANOVA followed by Bonferroni’s multiple-comparisons test. *p < 0.05, **p < 0.01, ***p < 0.001, ***p < 0.0001. Significant main effects and interaction effects indicated age-, sex-, and genotype-dependent modulation of hippocampal cytokine profiles in HLA-DRB115:01 mice.

Neuroinflammatory outcomes are shaped not only by cellular composition and immune trafficking but also by the local cytokine environment within the brain parenchyma. Given the age- and sex-dependent alterations in glial immune-associated responses, IL-3/IL-3R signaling, endothelial activation, and T cell accumulation observed in HLA-DRB1*15:01 mice, we next examined whether these cellular changes were accompanied by corresponding shifts in brain cytokine profiles. To address this, cytokine levels were quantified in lysed hippocampal tissue from WT and HLA mice across age and sex using a multiplex electrochemiluminescence assay (MSD). The cytokine panel included IFN-γ, IL-1β, IL-2, IL-4, IL-5, IL-6, IL-10, IL-12p70, KC/GRO, and TNF-α. Among the cytokines assessed, only IL-12p70, IL-10, and IL-2 exhibited detectable and statistically analyzable differences across age, sex, or genotype. Levels of IFN-γ, IL-1β, IL-4, IL-5, IL-6, KC/GRO, and TNF-α did not differ significantly between groups or were below the assay’s reliable detection range. Accordingly, subsequent analyses focused on IL-12p70, IL-10, and IL-2 (**Figure 9**).

Analysis of IL-12p70 levels in hippocampal lysates revealed age- and genotype-dependent differences specifically in female mice. In females, HLA mice exhibited significantly higher IL-12p70 levels at 9 months (p = 0.0002) and 15 months (p = 0.0065) compared with age-matched WT controls (**Figure 9A**). In addition, IL-12p70 levels in HLA females increased with age, with significantly higher levels at 9 and 15 months compared with 6 months (p = 0.02). In contrast, male mice showed no significant genotype- or age-dependent differences in IL-12p70 levels across the time points examined (**Figure 9A**).

Quantification of IL-10 levels revealed a strong genotype-dependent reduction in HLA mice, with clear sex-specific differences. In female mice, IL-10 levels were significantly lower in HLA animals than in age-matched WT controls at 9 months (p = 0.001) and 15 months (p = 0.025), whereas no significant difference was detected at 6 months (**Figure 9B**). In male mice, IL-10 levels were significantly lower in HLA animals than in WT controls at all ages examined (6, 9, and 15 months; all p < 0.0001) (**Figure 9B**). IL-10 levels did not exhibit significant age-dependent changes within either WT or HLA groups in females or males. Comparison across sexes further indicated that IL-10 levels were significantly lower in HLA males than in HLA females (**Figure 9B**).

Quantification of IL-2 levels in hippocampal lysates revealed significant genotype- and sex-dependent differences within the HLA genotype (**Figure 9C**). In female mice, IL-2 levels were consistently lower in HLA-DRB1*15:01 animals compared with age-matched WT controls at all ages examined (all p < 0.0001). In contrast, no significant genotype-dependent differences were detected in male mice. IL-2 levels did not show significant age-associated changes within either genotype in males or females. Comparison across sexes revealed a pronounced sex difference within the HLA genotype, with HLA males exhibiting significantly higher IL-2 levels than HLA females across ages (**Figure 9C**).

Together, these results indicate that HLA-DRB1*15:01 expression selectively alters the hippocampal cytokine environment in an age- and sex-dependent manner. Increased IL-12p70 in aged HLA females, combined with reduced IL-10 and suppressed IL-2 in HLA females, reflects a shift toward pro-inflammatory and dysregulated immune signaling within the aging brain. When considered alongside earlier findings of glial immune-associated changes, IL-3/IL-3R signaling, endothelial activation, and increased CD4^+^ T-cell accumulation, these cytokine changes support a coordinated neuroinflammatory phenotype associated with HLA-DRB1*15:01 during aging.

## 4 Discussion

The present study demonstrates that expression of the human autoimmune risk allele HLA-DRB1*15:01—a genetic risk factor for MS and susceptibility to late-onset neurodegenerative diseases —is associated with selective, age- and sex-dependent remodeling of the neuroimmune environment in the aging brain. By integrating behavioral analyses with flow cytometry, immunofluorescence, and hippocampal cytokine profiling, we identify a coordinated HLA-DRB1*15:01–associated phenotype characterized by microglial oxidative stress and immune-associated alterations, altered astrocyte–microglia immune signaling, endothelial activation at CNS interfaces, selective lymphoid cell accumulation, and targeted cytokine imbalance. Notably, these effects were most pronounced in aged females and coincided with the emergence of late-onset behavioral impairment, underscoring a female-biased neuroimmune vulnerability associated with HLA-DRB1*15:01. Although female mice exhibited age-associated reproductive/endocrine transitions, exploratory endocrine-stage stratification analyses did not reveal consistent endocrine-stage–dependent differences across the measured neuroimmune parameters, suggesting that the observed female-biased effects were more strongly associated with biological sex and aging than with reproductive cycling stage alone.

It is important to point out that in this study, HLA-DRB1*15:01 mice were not challenged with myelin peptides such as mMOG35–55 and did not develop clinical paralysis or overt autoimmune demyelination. In addition, this humanized model lacks endogenous murine MHC class II, thereby limiting classical murine MHC-II–dependent antigen presentation by CNS-resident and peripheral antigen-presenting cells (22). Consequently, the observed phenotypes reflect baseline, age-related neuroimmune alterations associated with HLA-DRB1*15:01, rather than effects of active autoimmune disease. At the same time, the absence of endogenous murine MHC-II represents an important caveat, as the immune architecture of this model is not identical to that of WT mice with intact murine MHC-II, and thus the phenotype should be interpreted as HLA-associated neuroimmune remodeling within an MHC-II–null context.

The cell-type specificity of HLA-DRB1*15:01 expression is also relevant when interpreting these findings. The chimeric HLA class II molecule is expressed under murine MHC class II regulatory control on an MHC class II–deficient background and is therefore expected to follow canonical MHC class II expression patterns, which under basal conditions are largely restricted to professional antigen-presenting cells, including dendritic cells, macrophages, and B cells. We did not directly assess cell-type-specific expression of HLA-DRB1*15:01 in non-classical MHC class II–expressing populations such as astrocytes, endothelial cells, or meningeal stromal cells. Accordingly, the alterations observed in these compartments cannot be attributed to direct transgene expression within those cell types. Rather, these findings are more appropriately interpreted as reflecting system-level neuroimmune alterations associated with HLA-DRB1*15:01–dependent immune signaling. Although MHC class II expression can be induced in non-professional cell types under inflammatory conditions, the present data do not support conclusions regarding cell-autonomous effects of HLA-DRB1*15:01 within CNS-resident populations.

Interpretation of allele-specific effects also requires consideration of this model’s targeted replacement design. The HLA-DRB1*15:01 mice differ from WT animals not only in MHC class II sequence, but also in the replacement of endogenous murine MHC-II with a human allele. Previous studies have shown that the presence or absence of murine MHC class II influences antigen presentation and CD4^+^ T-cell selection, thereby affecting downstream immune phenotypes (38, 39). Accordingly, the differences observed in this study reflect the neuroimmune landscape associated with HLA-DRB1*15:01 expression within a targeted replacement model relative to murine baseline conditions. Direct comparison across multiple human HLA class II alleles would further strengthen allele-specific interpretation; however, such approaches remain limited. Transgenic mouse models have been generated for a limited number of HLA class II alleles, including HLA-DRB1*01:01, which has been associated with lower risk or protective effects in multiple sclerosis (40–42). Additional alleles, such as HLA-DRB1*11, have also been implicated as protective through human genetic studies (42). However, available systems are not directly comparable, as they were developed using different genetic strategies and MHC class II backgrounds, both of which are known to influence immune phenotypes (41). Together, these limitations underscore the current lack of standardized *in vivo* systems for systematic comparison across risk and protective HLA class II alleles. Within this context, the present study provides an important framework for defining age- and sex-dependent neuroimmune features associated with HLA-DRB1*15:01 expression in a targeted replacement model. These findings show that expression of a disease-relevant human HLA allele is associated with substantial remodeling of the neuroimmune milieu under basal conditions and establish a foundation for future studies directly comparing multiple HLA alleles in controlled experimental systems. This is particularly relevant given the growing recognition that HLA class II variation contributes not only to autoimmune susceptibility, but also to broader neuroinflammatory and neurodegenerative processes.

A key finding of this study is the emergence of late-onset cognitive impairment restricted to aged female HLA-DRB1*15:01 mice. At 15 months of age, HLA females, but not males, exhibited deficits in nest building, recognition memory, and spatial discrimination, while WT mice of both sexes remained behaviorally intact. The delayed onset of impairment argues against early neurodevelopmental dysfunction and instead supports a model in which HLA-DRB1*15:01 interacts with aging-related processes, particularly in females. This sex bias aligns directionally with immune-mediated CNS disorders such as MS, in which sex and age shape susceptibility and disease trajectories (43, 44). Importantly, behavioral impairment temporally coincided with the strongest convergence of neuroimmune alterations, supporting the interpretation that functional decline arises within a progressively remodeled immune environment rather than from a single acute inflammatory trigger.

Microglial oxidative stress emerged as an early and robust feature of HLA-DRB1*15:01 expression. Both female and male HLA mice displayed age-dependent increases in MitoSOX-positive microglia beginning at midlife, whereas WT mice maintained low oxidative burden across ages. At advanced age, HLA females exhibited the highest levels of oxidative stress. Oxidative stress, defined as an imbalance between the production and clearance of reactive oxygen species, is closely linked to metabolic stress in microglia and can influence cellular homeostasis and immune responsiveness (45, 46). While oxidative stress alone cannot be interpreted as definitive evidence of microglial priming, accumulating evidence indicates that an altered redox state can modulate microglial functional properties and responsiveness to subsequent immune cues (47–49). In this context, the emergence of elevated oxidative stress at 9 months, preceding behavioral impairment at 15 months in females, suggests that redox imbalance represents an early alteration in microglial state, rather than a downstream consequence of behavioral decline. Because microglial oxidative stress emerged at midlife, ahead of behavioral impairment and immune cell accumulation, it may reflect an early permissive condition that influences later neuroimmune remodeling.

Consistent with the increased microglial oxidative stress observed in HLA-DRB1*15:01 mice, expression of the microglial immune-associated markers CD14 and CD68 also increased in an age- and sex-dependent manner. These markers were assessed using MHC-II–independent readouts, an approach required by the absence of endogenous murine MHC class II in this model and which allowed genotype comparisons without reliance on MHC-II expression. Increased CD14 expression is consistent with heightened innate immune responsiveness (35, 36), whereas elevated CD68 levels reflect enhanced lysosomal and phagolysosomal activity, commonly associated with reactive microglial states (37). Although both male and female HLA mice exhibited age-related increases in these markers, the strongest elevations were generally observed in aged female HLA mice. Future studies incorporating additional aging- and disease-associated microglial markers, including Clec7a and CD11c/Itgax, may help further define whether the HLA-associated microglial phenotype overlaps with established neurodegenerative or aging-related microglial programs (33, 50).

At the tissue level, hippocampal immunofluorescence revealed alterations in microglial morphometric features, including reduced branching complexity and shorter branch length in HLA mice relative to WT controls. Notably, overall IBA1^+^ area showed only modest differences between groups, suggesting that the observed phenotype primarily reflected altered microglial organization and morphology rather than major expansion of the total microglial population. Under homeostatic conditions, ramified microglia exhibit highly dynamic process motility and continuously survey neuronal somata and synapses, a defining feature of homeostatic microglial function. In contrast, chronic neurodegenerative and inflammatory conditions are frequently associated with reduced ramification and altered microglial–neuronal interactions (51–53). However, microglial morphology alone is insufficient to define a specific activation state, as microglia exhibit substantial structural and functional heterogeneity across physiological and pathological contexts (50). In the present study, the altered morphometric phenotype was accompanied by increased oxidative stress and elevated expression of immune-associated markers, including CD68 and CD14. Together, these findings support a broader shift toward a metabolically stressed and immune-associated microglial state linked to HLA-DRB1*15:01 expression that emerges progressively with aging rather than an isolated structural change.

A mechanistically related aspect of this study is the coordinated, age- and sex-dependent amplification of the IL-3/IL-3 receptor axis across astrocytes and microglia. IL-3R expression on microglia increased with age in both genotypes but was markedly elevated in HLA-DRB1*15:01 mice, with the strongest induction observed in aged females. Notably, MS exhibits a strong female bias, and the IL3RA gene is encoded on the X chromosome (54), raising the possibility that sex-linked regulatory mechanisms may contribute to the enhanced IL-3R expression observed in HLA females. In parallel, astrocytic IL-3 expression increased selectively in HLA mice, again with the greatest magnitude in females. This coordinated pattern is notable because accumulating evidence indicates that IL-3 functions as a local CNS immune-modulatory signal, rather than a systemic hematopoietic cytokine, shaping microglial immune tone, chemotactic programs, and regulated immune recruitment at CNS interfaces (55–62). Importantly, astrocytes have been identified as sources of IL-3 in both healthy and inflamed CNS tissue, and IL-3 expression is not linked to classical astrogliosis (57, 59). Consistent with these observations, increased IL-3 immunoreactivity in HLA mice occurred without generalized GFAP upregulation, supporting selective astrocyte functional remodeling rather than generalized astrogliosis.

Interpretation of the microglial findings also requires consideration of the distinct spatial scales captured by whole-brain flow cytometry and hippocampal immunofluorescence. Flow cytometry provided a global assessment of CNS immune remodeling across dissociated brain tissue, whereas immunofluorescence enabled regional evaluation of hippocampal cellular organization and local neuroimmune interactions. Accordingly, some features showed directional consistency across approaches, including enhanced IL-3R–associated signaling within microglial populations in HLA mice, while others, particularly CD68-related changes, appeared more regionally heterogeneous at the tissue level. These differences likely reflect localized microenvironmental remodeling within vulnerable hippocampal regions rather than direct inconsistency between methodologies.

The functional consequences of IL-3 signaling are highly context dependent. In Alzheimer’s disease, IL-3 promotes microglial clustering and clearance of amyloid and tau, exerting protective effects, whereas in human MS and an EAE murine model, IL-3 signaling exacerbates disease by promoting immune recruitment and neuroinflammation (59, 63). These contrasting outcomes underscore that the impact of IL-3/IL-3R signaling depends on cellular targets, disease environment, and immune architecture. In the present study, IL-3/IL-3R amplification emerged in the absence of EAE induction, supporting the concept that genetic risk conferred by HLA-DRB1*15:01 expression may modulate IL-3–mediated signaling as part of baseline, age-associated immune tuning within the CNS, rather than as a secondary consequence of overt inflammatory disease. Nevertheless, these findings remain correlative. While the close coupling between astrocytic IL-3 expression, microglial IL-3R upregulation, and immune-associated microglial changes strongly implicates this axis in shaping the observed phenotype, direct causality cannot be established without targeted genetic or pharmacological manipulation.

Despite the absence of overt tissue pathology, HLA-DRB1*15:01 expression was associated with coordinated structural and organizational alterations in hippocampal organization at advanced age, including disrupted myelin and axonal organization and increased microglial localization within regions of altered tissue structure. The more pronounced alterations observed in females paralleled the stronger immune phenotype in this group. Together, these findings support the concept that chronic HLA-DRB1*15:01–associated neuroimmune remodeling is accompanied by localized alterations in hippocampal tissue organization during aging.

HLA-DRB1*15:01 also influenced immune dynamics at CNS borders. Rather than broad infiltration, we observed selective accumulation of CD4^+^ T cells and NKT cells in the meninges and brain, with greater accumulation observed in females. Endothelial activation was also observed, characterized by increased expression of ICAM-1 and E-selectin. Together, these findings suggest a state of endothelial priming sufficient to support regulated leukocyte recruitment, without evidence of widespread vascular inflammatory activation. In addition, the evidence that IL-3/IL-3Rα signaling can drive chemotactic programming in myeloid cells and promote CNS immune recruitment (59) raises the possibility that IL-3/IL3R signaling contributes to immune activity at CNS borders in HLA-DRB1*15:01 mice.

Finally, hippocampal cytokine profiling reinforced the theme of selectivity. Among the 10 cytokines assessed, only IL-12p70, IL-10, and IL-2 showed consistent differences across age, sex, and genotype, indicating a selective alteration of cytokine signaling rather than a global inflammatory response. The cytokine profile observed in aged female HLA mice, elevated IL-12p70 coupled with reduced IL-10 and IL-2, suggests a shift toward pro-inflammatory immune polarization with diminished regulatory balance, rather than generalized cytokine excess. Because cytokine measurements were derived from whole hippocampal lysates, cellular sources cannot be resolved, underscoring the need for future spatial and cell-specific analyses.

## 5 Conclusions

Taken together, these findings support an integrated model in which HLA-DRB1*15:01 lowers the threshold for age-related neuroimmune dysregulation rather than directly inducing autoimmune demyelination. In the absence of antigenic challenge, this allele is associated with a coordinated, sex-biased phenotype involving oxidative stress, microglial immune-associated and morphometric alterations, selective IL-3/IL-3R signaling, endothelial priming, compartmentalized immune recruitment, and targeted cytokine imbalance. This study demonstrates that these neuroimmune alterations emerge progressively during basal aging and converge across multiple levels of analysis in parallel with female-biased behavioral impairment. At the same time, interpretation must be tempered by key limitations, including the absence of murine MHC-II, the correlative nature of IL-3/IL-3R associations, and the qualitative nature of some tissue-level findings. Future studies incorporating pathway perturbation, spatial transcriptomics, inflammatory or antigenic challenges, and additional comparative HLA class II models will be important for defining how these baseline neuroimmune alterations interact with autoimmune mechanisms during disease initiation and progression.

## Data Availability

The raw data supporting the conclusions of this article will be made available by the authors, without undue reservation.
