# Supplementary material for: HLA-DRB1*15:01 drives sex- and age-dependent microglial immune phenotypes and neuroimmune signaling
Source: Front Immunol. 2026 Jun 16;17:1796692. doi: 10.3389/fimmu.2026.1796692 (PMC13314512; doi:10.3389/fimmu.2026.1796692)
Supplement: Supplementary file 1 [file Presentation1.pptx]

## Slide 1
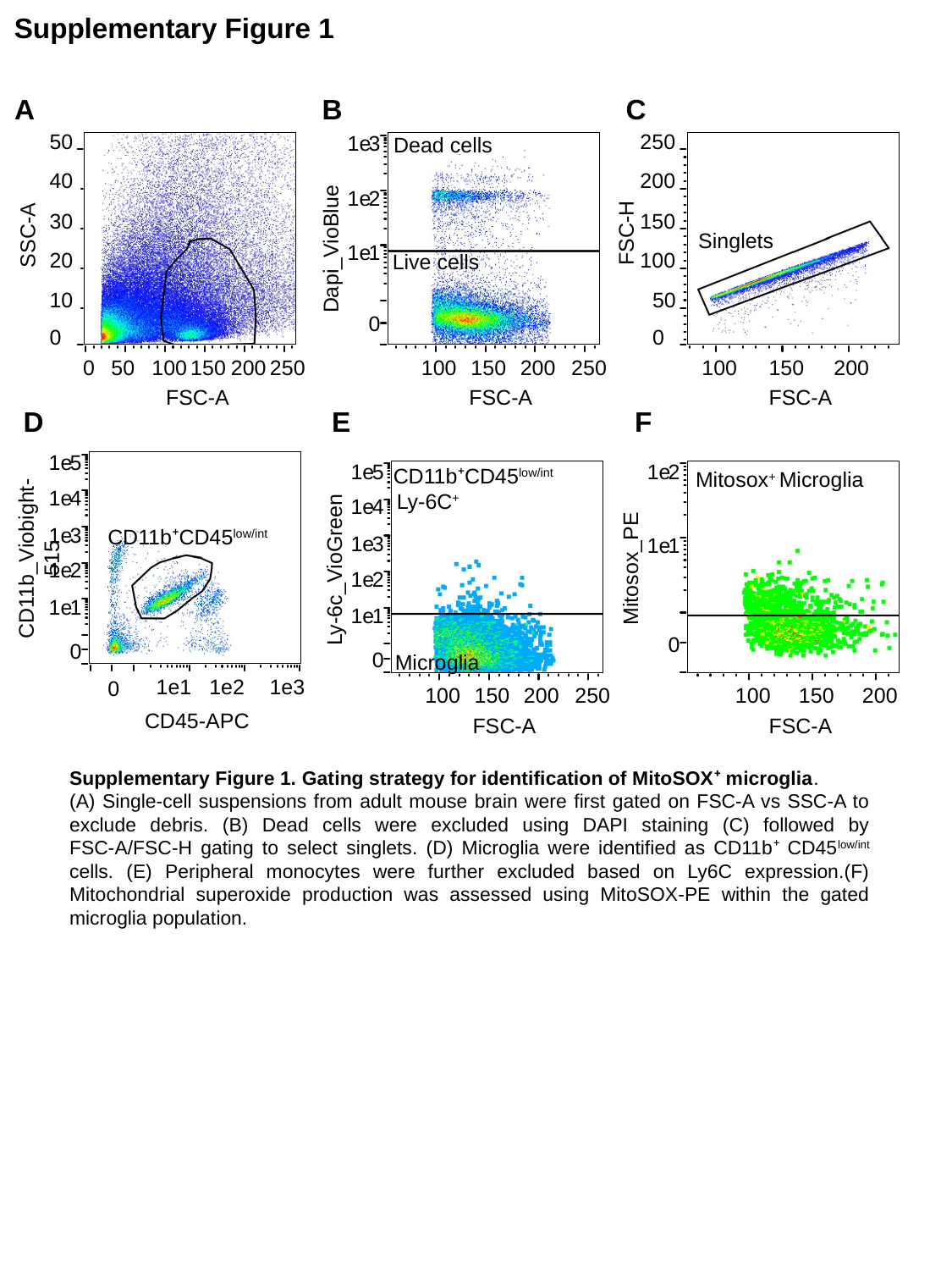

Supplementary Figure 1
A
B
C
250
200
FSC-H
150
100
50
0
100
150
200
FSC-A
Singlets
50
40
30
SSC-A
20
10
0
0
50
100
150
200
1e
3
1e
2
Dapi_VioBlue
1e
1
0
100
150
200
250
FSC-A
Dead cells
Live cells
250
FSC-A
D
E
F
1e
5
1e
4
1e
3
CD11b_Viobight-515
1e
2
1e
1
0
0
CD45-APC
1e1
1e2
1e3
1e
5
CD11b⁺CD45low/int
 Ly-6C+
1e
4
1e
3
Ly-6c_VioGreen
1e
2
1e
1
0
Microglia
100
150
200
250
FSC-A
1e
2
Mitosox+ Microglia
1e
1
Mitosox_PE
0
100
150
200
FSC-A
CD11b⁺CD45low/int
Supplementary Figure 1. Gating strategy for identification of MitoSOX⁺ microglia.
(A) Single-cell suspensions from adult mouse brain were first gated on FSC-A vs SSC-A to exclude debris. (B) Dead cells were excluded using DAPI staining (C) followed by FSC-A/FSC-H gating to select singlets. (D) Microglia were identified as CD11b⁺ CD45low/int cells. (E) Peripheral monocytes were further excluded based on Ly6C expression.(F) Mitochondrial superoxide production was assessed using MitoSOX-PE within the gated microglia population.

## Slide 2
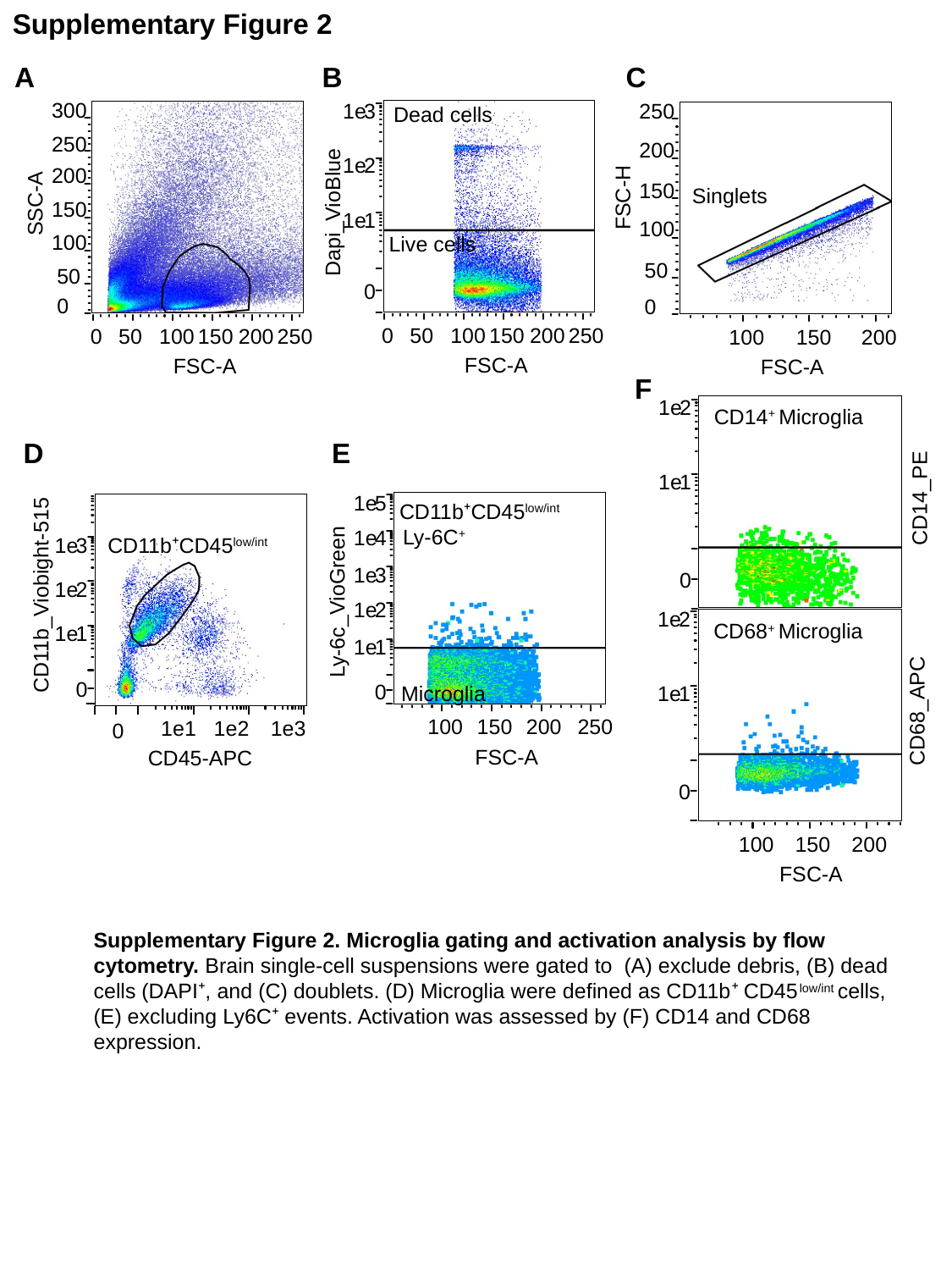

Supplementary Figure 2
A
B
C
300
250
SSC-A
200
150
100
50
0
0
50
100
150
200
250
FSC-A
1e
3
Dead cells
1e
2
Dapi_VioBlue
1e
1
Live cells
0
0
50
100
150
200
250
FSC-A
250
200
150
Singlets
FSC-H
100
50
0
100
150
200
FSC-A
F
1e
1e
2
1
0
CD14+ Microglia
CD14_PE
1e
1e
2
1
0
CD68+ Microglia
CD68_APC
100
150
200
FSC-A
D
E
1e
3
CD11b⁺CD45low/int
1e
2
CD11b_Viobight-515
1e
1
0
1e1
1e2
0
1e3
CD45-APC
1e
5
1e
4
1e
3
Ly-6c_VioGreen
1e
2
1e
1
0
100
150
200
250
FSC-A
CD11b⁺CD45low/int
 Ly-6C+
Microglia
Supplementary Figure 2. Microglia gating and activation analysis by flow cytometry. Brain single-cell suspensions were gated to (A) exclude debris, (B) dead cells (DAPI⁺, and (C) doublets. (D) Microglia were defined as CD11b⁺ CD45low/int cells, (E) excluding Ly6C⁺ events. Activation was assessed by (F) CD14 and CD68 expression.

## Slide 3
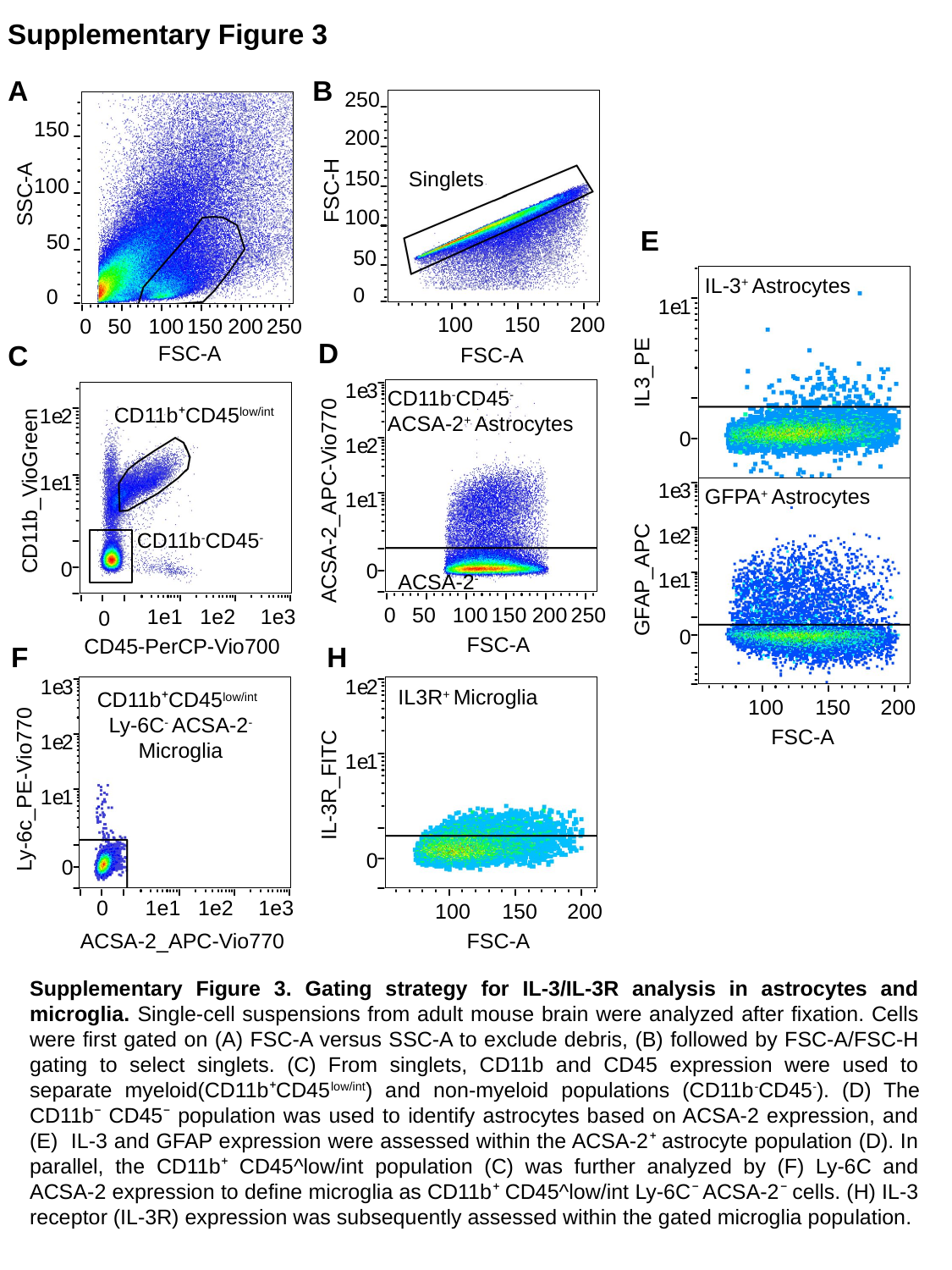

Supplementary Figure 3
250
200
FSC-H
150
100
50
0
100
150
200
FSC-A
150
SSC-A
100
50
0
0
50
100
150
200
250
FSC-A
A
B
Singlets
E
1e
1
IL3_PE
0
1e
3
1e
2
1e
1
GFAP_APC
0
100
150
200
FSC-A
IL-3+ Astrocytes
D
C
1e
3
1e
2
1e
1
ACSA-2_APC-Vio770
0
0
50
100
150
200
250
FSC-A
CD11b-CD45-
ACSA-2+ Astrocytes
1e
2
1e
1
CD11b_VioGreen
0
1e1
1e2
0
1e3
CD45-PerCP-Vio700
CD11b⁺CD45low/int
GFPA+ Astrocytes
CD11b-CD45-
ACSA-2-
F
H
1e
3
1e
2
Ly-6c_PE-Vio770
1e
1
0
ACSA-2_APC-Vio770
1e1
1e2
1e3
0
1e
2
1e
1
IL-3R_FITC
0
100
150
200
FSC-A
IL3R+ Microglia
CD11b⁺CD45low/int
 Ly-6C- ACSA-2-
Microglia
Supplementary Figure 3. Gating strategy for IL-3/IL-3R analysis in astrocytes and microglia. Single-cell suspensions from adult mouse brain were analyzed after fixation. Cells were first gated on (A) FSC-A versus SSC-A to exclude debris, (B) followed by FSC-A/FSC-H gating to select singlets. (C) From singlets, CD11b and CD45 expression were used to separate myeloid(CD11b⁺CD45low/int) and non-myeloid populations (CD11b-CD45-). (D) The CD11b⁻ CD45⁻ population was used to identify astrocytes based on ACSA-2 expression, and (E) IL-3 and GFAP expression were assessed within the ACSA-2⁺ astrocyte population (D). In parallel, the CD11b⁺ CD45^low/int population (C) was further analyzed by (F) Ly-6C and ACSA-2 expression to define microglia as CD11b⁺ CD45^low/int Ly-6C⁻ ACSA-2⁻ cells. (H) IL-3 receptor (IL-3R) expression was subsequently assessed within the gated microglia population.

## Slide 4
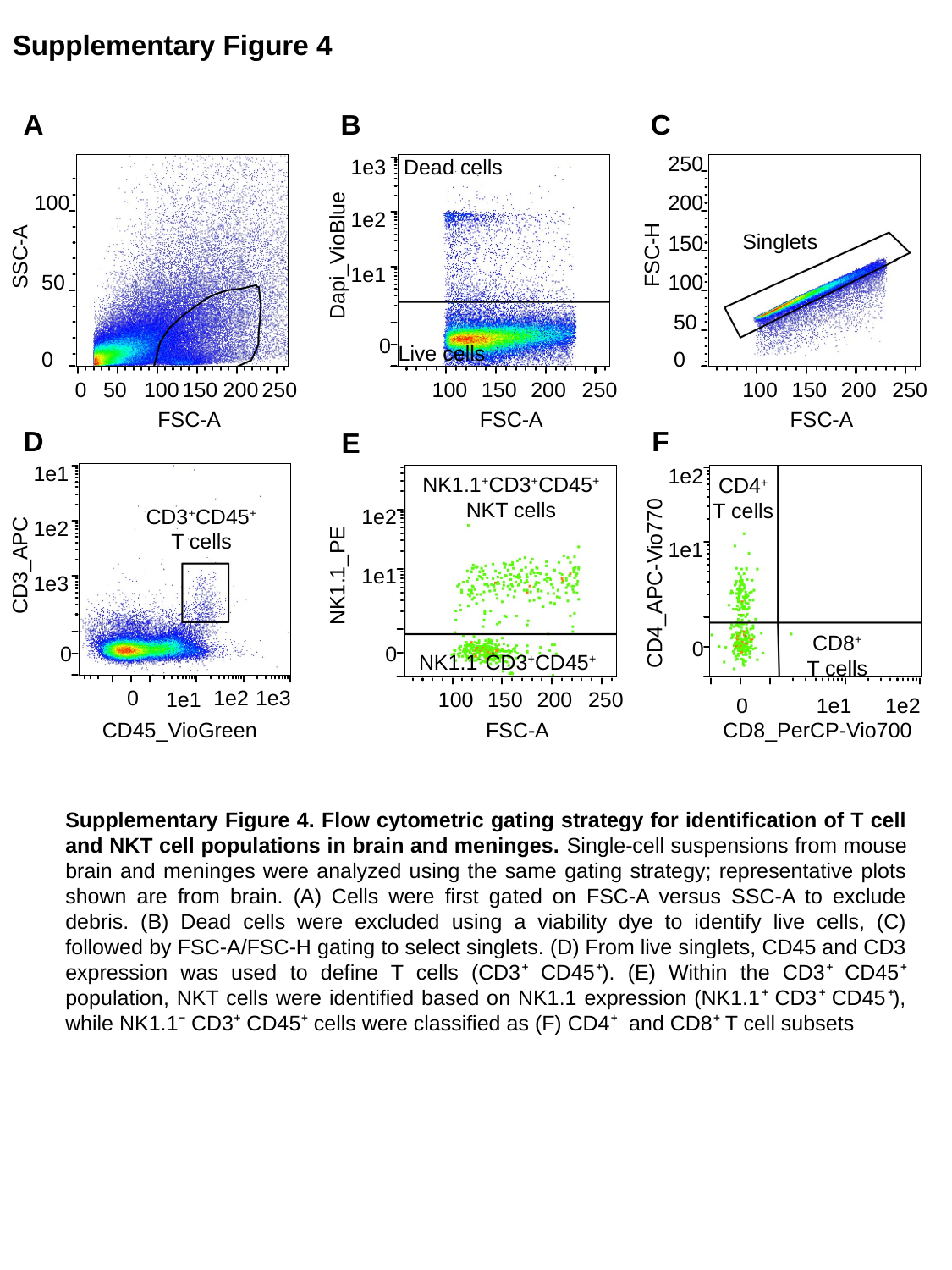

Supplementary Figure 4
A
B
C
250
200
FSC-H
150
100
50
0
100
150
200
250
FSC-A
100
SSC-A
50
0
0
50
100
150
200
250
FSC-A
1e3
1e2
Dapi_VioBlue
1e1
0
100
150
200
250
FSC-A
Dead cells
Singlets
Live cells
D
F
E
1e1
1e2
CD3_APC
1e3
0
0
1e2
1e3
1e1
CD45_VioGreen
1e2
1e1
CD4_APC-Vio770
0
0
1e1
1e2
CD8_PerCP-Vio700
NK1.1+CD3+CD45+
NKT cells
1e2
1e1
NK1.1_PE
0
100
150
200
250
FSC-A
CD4+
T cells
CD3+CD45+
T cells
CD8+
T cells
NK1.1- CD3+CD45+
Supplementary Figure 4. Flow cytometric gating strategy for identification of T cell and NKT cell populations in brain and meninges. Single-cell suspensions from mouse brain and meninges were analyzed using the same gating strategy; representative plots shown are from brain. (A) Cells were first gated on FSC-A versus SSC-A to exclude debris. (B) Dead cells were excluded using a viability dye to identify live cells, (C) followed by FSC-A/FSC-H gating to select singlets. (D) From live singlets, CD45 and CD3 expression was used to define T cells (CD3⁺ CD45⁺). (E) Within the CD3⁺ CD45⁺ population, NKT cells were identified based on NK1.1 expression (NK1.1⁺ CD3⁺ CD45⁺), while NK1.1⁻ CD3⁺ CD45⁺ cells were classified as (F) CD4⁺ and CD8⁺ T cell subsets

## Slide 5
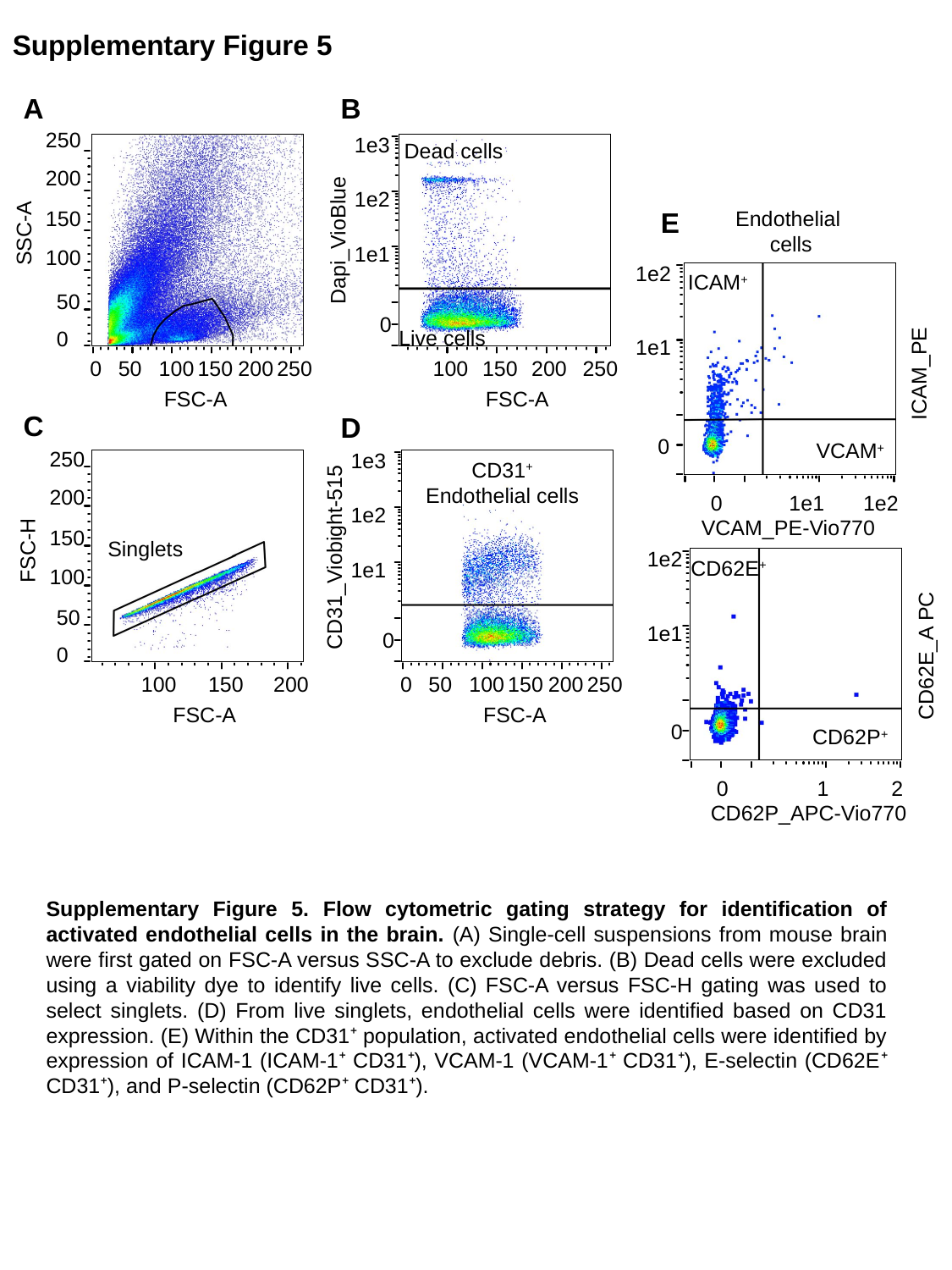

Supplementary Figure 5
A
B
250
200
SSC-A
150
100
50
0
0
50
100
150
200
250
FSC-A
1e3
1e2
Dapi_VioBlue
1e1
0
100
150
200
250
FSC-A
Dead cells
E
Endothelial
 cells
1e2
1e1
ICAM_PE
0
0
1e1
1e2
VCAM_PE-Vio770
ICAM+
Live cells
C
D
250
200
FSC-H
150
100
50
0
100
150
200
FSC-A
VCAM+
1e3
1e2
CD31_Viobight-515
1e1
0
0
50
100
150
200
250
FSC-A
CD31+
Endothelial cells
Singlets
1e2
1e1
CD62E_A PC
0
0
1
2
CD62P_APC-Vio770
CD62E+
CD62P+
Supplementary Figure 5. Flow cytometric gating strategy for identification of activated endothelial cells in the brain. (A) Single-cell suspensions from mouse brain were first gated on FSC-A versus SSC-A to exclude debris. (B) Dead cells were excluded using a viability dye to identify live cells. (C) FSC-A versus FSC-H gating was used to select singlets. (D) From live singlets, endothelial cells were identified based on CD31 expression. (E) Within the CD31⁺ population, activated endothelial cells were identified by expression of ICAM-1 (ICAM-1⁺ CD31⁺), VCAM-1 (VCAM-1⁺ CD31⁺), E-selectin (CD62E⁺ CD31⁺), and P-selectin (CD62P⁺ CD31⁺).

## Slide 6
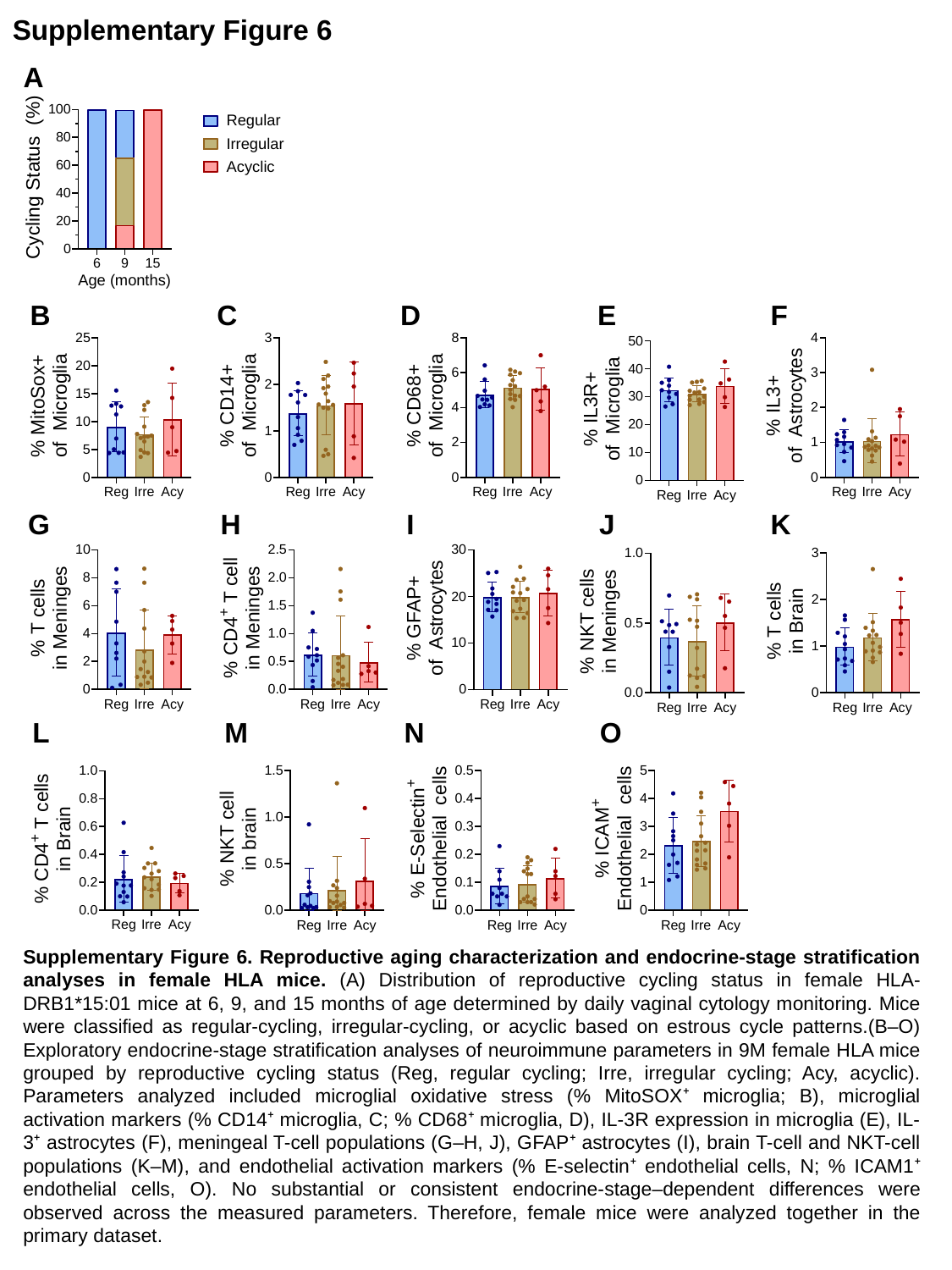

Supplementary Figure 6
A
B
C
D
E
F
G
H
I
J
K
L
M
N
O
Supplementary Figure 6. Reproductive aging characterization and endocrine-stage stratification analyses in female HLA mice. (A) Distribution of reproductive cycling status in female HLA-DRB1*15:01 mice at 6, 9, and 15 months of age determined by daily vaginal cytology monitoring. Mice were classified as regular-cycling, irregular-cycling, or acyclic based on estrous cycle patterns.(B–O) Exploratory endocrine-stage stratification analyses of neuroimmune parameters in 9M female HLA mice grouped by reproductive cycling status (Reg, regular cycling; Irre, irregular cycling; Acy, acyclic). Parameters analyzed included microglial oxidative stress (% MitoSOX⁺ microglia; B), microglial activation markers (% CD14⁺ microglia, C; % CD68⁺ microglia, D), IL-3R expression in microglia (E), IL-3⁺ astrocytes (F), meningeal T-cell populations (G–H, J), GFAP⁺ astrocytes (I), brain T-cell and NKT-cell populations (K–M), and endothelial activation markers (% E-selectin⁺ endothelial cells, N; % ICAM1⁺ endothelial cells, O). No substantial or consistent endocrine-stage–dependent differences were observed across the measured parameters. Therefore, female mice were analyzed together in the primary dataset.
